# Mathematical modelling of human P2X-mediated plasma membrane electrophysiology and calcium dynamics in microglia

**DOI:** 10.1371/journal.pcbi.1009520

**Published:** 2021-11-01

**Authors:** Alireza Poshtkohi, John Wade, Liam McDaid, Junxiu Liu, Mark Dallas, Angela Bithell

**Affiliations:** 1 School of Computing, Engineering and Intelligent Systems, Ulster University, Londonderry, United Kingdom; 2 School of Pharmacy, University of Reading, Reading, United Kingdom; Weill Medical College of Cornell University, UNITED STATES

## Abstract

Regulation of cytosolic calcium (Ca^2+^) dynamics is fundamental to microglial function. Temporal and spatial Ca^2+^ fluxes are induced from a complicated signal transduction pathway linked to brain ionic homeostasis. In this paper, we develop a novel biophysical model of Ca^2+^ and sodium (Na^+^) dynamics in human microglia and evaluate the contribution of purinergic receptors (P2XRs) to both intracellular Ca^2+^ and Na^+^ levels in response to agonist/ATP binding. This is the first comprehensive model that integrates P2XRs to predict intricate Ca^2+^ and Na^+^ transient responses in microglia. Specifically, a novel compact biophysical model is proposed for the capture of whole-cell patch-clamp currents associated with P2X_4_ and P2X_7_ receptors, which is composed of only four state variables. The entire model shows that intricate intracellular ion dynamics arise from the coupled interaction between P2X_4_ and P2X_7_ receptors, the Na^+^/Ca^2+^ exchanger (NCX), Ca^2+^ extrusion by the plasma membrane Ca^2+^ ATPase (PMCA), and Ca^2+^ and Na^+^ leak channels. Both P2XRs are modelled as two separate adenosine triphosphate (ATP) gated Ca^2+^ and Na^+^ conductance channels, where the stoichiometry is the removal of one Ca^2+^ for the hydrolysis of one ATP molecule. Two unique sets of model parameters were determined using an evolutionary algorithm to optimise fitting to experimental data for each of the receptors. This allows the proposed model to capture both human P2X_7_ and P2X_4_ data (hP2X_7_ and hP2X_4_). The model architecture enables a high degree of simplicity, accuracy and predictability of Ca^2+^ and Na^+^ dynamics thus providing quantitative insights into different behaviours of intracellular Na^+^ and Ca^2+^ which will guide future experimental research. Understanding the interactions between these receptors and other membrane-bound transporters provides a step forward in resolving the qualitative link between purinergic receptors and microglial physiology and their contribution to brain pathology.

## Author summary

Mathematical modelling and computer simulation are powerful tools by which we can analyse complex biological systems, particularly, neural phenomena involved in brain dysfunction. In this research, we develop a theoretical foundation for studying P2X-mediated calcium and sodium signalling in human microglial cells. Microglia, which are brain-resident macrophages, restructure their intracellular actin cytoskeleton to enable motility; this restructuring requires a complex molecular cascade involving a set of ionic channels, membrane-coupled receptors and cytosolic components. Recent studies highlight the importance for increasing our understanding of microglia physiology, since their functions play critical roles in both normal physiological and pathological dynamics of the brain. There is a need to develop reliable human cellular models to investigate the biology of microglia aimed at understanding the influence of purinergic signalling in brain dysfunction to provide novel drug discovery targets. In this work, a detailed mathematical model is built for the dynamics of human P2XRs in microglia. Subsequently, experimental whole-cell currents are used to derive P2X-mediated electrophysiology of human microglia (i.e. sodium and calcium dynamics, and membrane potential). Our predictions reveal new quantitative insights into P2XRs on how they regulate ionic concentrations in terms of physiological interactions and transient responses.

## Introduction

Recently, microglia have attracted wide attention owing to their ability to undergo a variety of morphological configurations in health and disease [1–3]. Motile microglial cells play a key defensive role in the central nervous system (CNS) through their role in clearing pathogens and active maintenance of neurons and synapses. Microglia appearance can range from ramified to amoeboid-like cells dependent on their microenvironment [4,5], however this two state model does not capture the full range of microglia heterogenity [6]. Restructuring of the actin cytoskeleton for directed motility in microglia consists of a complex molecular cascade that involves several membrane-coupled receptors [5]. These receptors enable the microglia to detect subtle concentration changes in their environment in order to begin either process extension or whole-cell chemotaxis. Three major families of purinergic receptors are found in human, rat and mouse microglia. These include adenosine receptors (A_1_, A_2A_, A_2B_ and A_3_), P2X receptors (P2XRs) (P2X_1_, P2X_4_ and P2X_7_) and P2Y receptors (P2Y_2_, P2Y_6_ and P2Y_12-14_) [7]. Evidence to date suggests that P2X_4,_ P2X_7_ and P2Y_12_ are the most important receptors for microglial directed motility both *in vitro* and *in vivo*, where spontaneous calcium (Ca^2+^) transients play a key role [8–12]. P2XRs are plasma membrane trimeric assemblies, which bind ATP for ionic permeation through the plasma membrane. One of the important elements in controlling intracellular calcium ([Ca_i_^2+^]) is ATP and its activation of purinergic P2XRs in eukaryotic cells [13,14]. Upon adenosine triphosphate (ATP) binding, a channel pore opens for Ca^2+^ and other ionic currents to translocate into the cell. Electrophysiology recordings are used to study the P2XR biophysical properties in response to ATP stimualtion. The main goal of this paper is to develop a biologically faithful model of ATP-triggered P2XR Ca^2+^ and sodium (Na^+^) signalling transduction and plasma membrane electrophysiology in microglia.

Microglia are involved in many neurodegenerative disorders, such as Parkinson’s and Alzheimer’s diseases, and are massively activated during brain injuries such as stroke [3,15,16]. There are many known hurdles to overcome in the study of microglia for drug discovery strategies [17–19]. Robust and scalable cellular models have to be built that help understand the biology of microglia for drug development. To the best of our knowledge, there is no theoretical work and no successful experimental study of simultaneous activation of several ionotropic receptors in human microglia. There is substantial evidence that both P2X_4_ and P2X_7_ receptors are expressed in microglial cells [7,20,21]. However, human microglia experiments failed to isolate fucntional P2X_4_ receptors [22], therefore a modelling approach will deepen our understanding of the contribution of each of these receptors to Ca^2+^ and Na^+^ dynamics within human microglia. Moreover, our model predictions for human microglia and the comparison of the kink-like behaviour of their responses confirm that intricate intracellular Ca^2+^ transients arise from a complex cooperative activation of both P2XRs and other ionic extruders and pumps. It is anticipated that the future *in situ* and *in vivo* experiments relative to human microglia research will be able to validate the theoretical findings supported by faithful biological inferences made in this paper. There is a high demand to develop reliable cellular human models where microglia can be studied with the aim of furthering our understanding of neuroglia interactions.

## Methods

**Fig 1:**
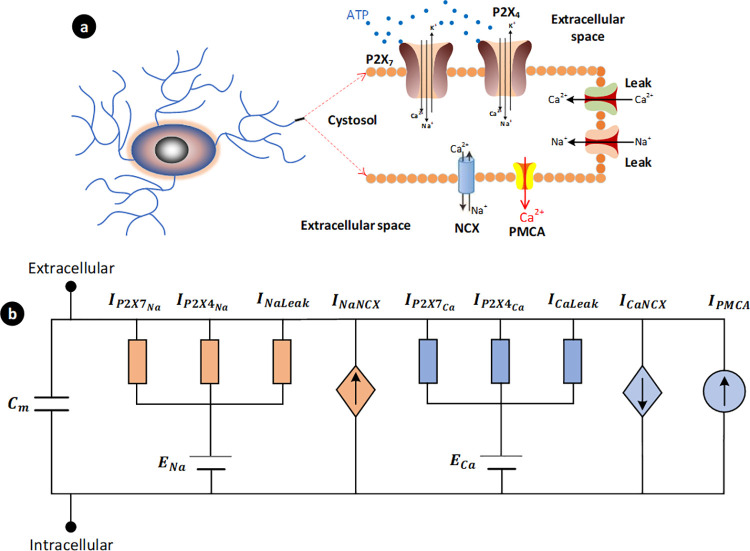
P2X-mediated Ca^2+^ signalling network developed for this study involved in microglial directed motility. The computational model is classified into two parts: (a) fluid compartmental model and (b) microglial membrane electrophysiology. The PMCA (*I*_*PMCA*_) and NCX (in the forward mode) remove cytosolic Ca^2+^. Cellular electrophysiology is included in our model by involving the transmembrane currents that underpin P2X-mediated Ca^2+^ signalling and microglia homeostasis. The P2X currents for each receptor are modelled as two individual Na^+^ and Ca^2+^ conductance-based channels ($I_{{P2X}_{Na}}$ and $I_{{P2X}_{Ca}}$), and both Na^+^ and Ca^2+^ leak channels (*I*_*NaLeak*_ and *I*_*CaLeak*_) are also included in the proposed model; the dynamic behaviour of the reversal potentials (*E*_*Na*_ and *E*_*Ca*_) are also taken into account. The current through the NCX channel is divided into a sodium current (*I*_*NaNCX*_) and a calcium current (*I*_*CaNCX*_).

The high-level view of the biophysical model is shown in Fig 1, which includes P2XRs, the Na^+^/Ca^2+^ exchanger (NCX) and the plasma membrane Ca^2+^ ATPase (PMCA) along with Ca^2+^ and Na^+^ leakage channels. Microglia ionic homeostasis is both dynamic and complex, involving a myriad of receptors, effectors, proteins, channels and processes. The proposed model for intracellular Ca^2+^ and Na^+^ dynamics mediated by P2XRs is based on the mass action kinetics formulated by four state variables to capture the complex mechanics of both P2X_4_ and P2X_7_ receptors when activated by ATP.

Increases in extracellular ATP levels (e.g., from damaged neurons) are detected by microglia and they extend their processes towards the elevated ATP levels through chemotaxis principles [7]. Fig 1A summarises the key intracellular signalling transduction pathways for this purpose. The two P2XRs bind ATP and change their conformation, allowing a rapid rise in intracellular [Ca^2+^] through Ca^2+^ influx. Published experimental data for hP2XRs obtained from both native preparations and expression systems were employed throughout the model development [22,23]. While no data for hP2X_4_R currently exists for microglia, this paper uses functional hP2X_4_ data recorded in expression systems [23]. Given the overexpression of receptor protein and the magnitude of expression system currents, the data has been scaled to better align with native microglial P2X currents [24] (in the order of pA).

### P2X kinetic models

P2XRs work as cation channels and open after ATP binding. They allow rapid entrance of Na^+^ and Ca^2+^ ions into the cell with an efflux of potassium (K^+^): note that it has recently been proposed that K^+^ efflux is primarily driven by the TWIK2 K^+^ channel [25] but this mechanism needs further investigation, and therefore K^+^ dynamics are not considered in this article. Both P2X_4_ and P2X_7_ receptors in microglia share similar trimeric configurations but display different biophysical properties. P2X_7_ receptors in the CNS can activate the proliferation of microglia, modulate cell phagocytosis and induce TNF-α production [7]. The P2X_4_ receptor, which is stimulated at micromolar ranges of ATP, induces an instantaneous peak current and desensitises within seconds [23,24]. In contrast, millimolar ATP triggers P2X_7_ receptors and maintains ionic currents during the ATP application [22].

Biophysically different Markov models have been widely used to describe the binding sites of P2XRs in whole-cell current configurations with eight or more state variables. In this work a new mathematical model was developed that avoids the complex Markov approach [26–28]. It is worth noting that eight-state Markov models [29,30] were not successful in providing a good fit to the human data in [22,23] (see S2 Text). Therefore, we developed a minimal model for P2XRs as follows.

**Fig 2:**
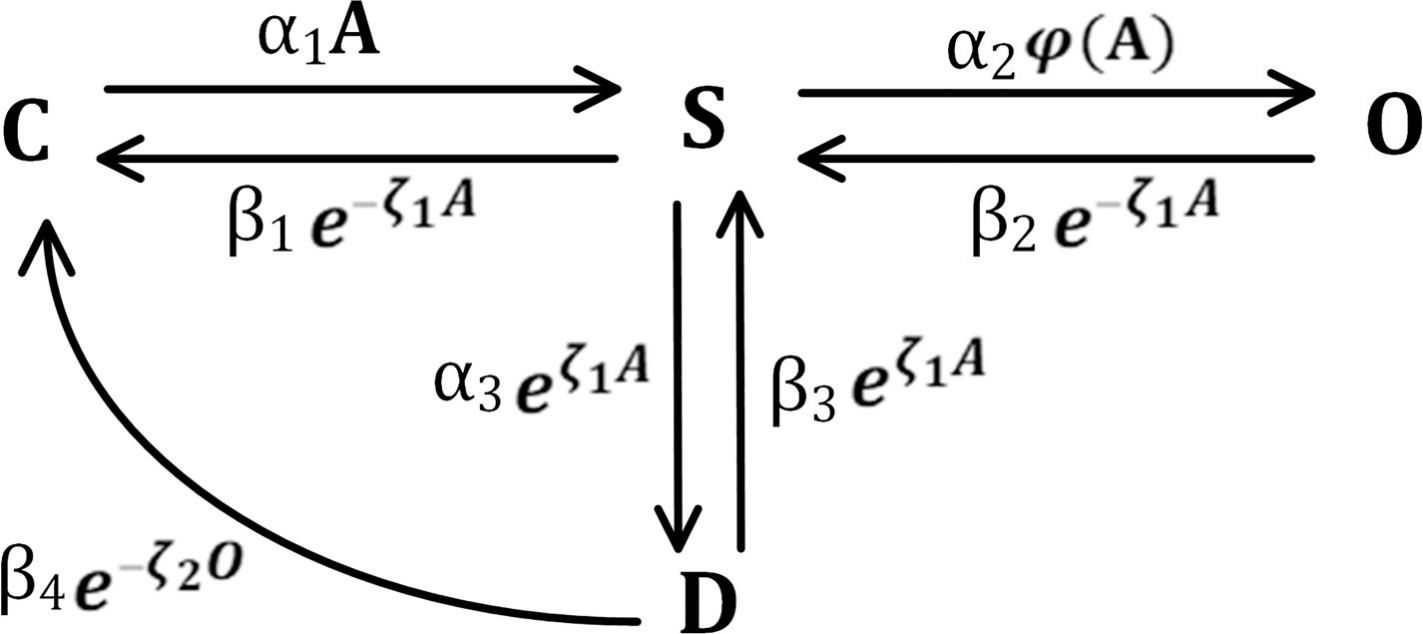
The biophysical P2X model: a compact kinetic reaction network which captures the total current induced by microglial hP2X_7_ or hP2X_4_ receptors dependant on the model parameters used. The agonist is denoted by the letter *A*. The states C, S, D and O respectively stand for *closed*, *sensitised*, *desensitised* and *opened*. The α and β parameters are the forward and reverse kinetic rates. The rate function *φ* between S and O is expressed as *φ*(A) = *A* for the hP2X_7_ receptor and $\varphi (A)=e^{\zeta_{1}A}$ for the hP2X_4_ receptor.

Fig 2 shows a biochemical kinetic reaction network that underpins both the hP2X_7_ and hP2X_4_ receptors comprising of only four state variables (where C, S, D and O states represent *closed*, *sensitised*, *desensitised* and *opened* respectively). By introducing variable exponential kinetic rates into this four-variable model (inpisred by other similar biophysical models [31]) in a similar way to the approach of Hodgkin and Huxley (HH) [32], the model becomes a less complex realisation as compared to the existing P2X Markov models, which is capable of capturing the major gating properties of microglial P2XRs. Note that the HH model benefits from voltage-gated exponential rates while the model herein makes use of agonist-gated exponential rates. The gating properties of the P2X channels are broken into four individual stages, including: activation (S → O) and sensitisation (C → S and D → S), desensitisation (S → D and then D → C) and deactivation (from O to S and then C, or S to D and then to C). Activation is a quick process, which happens after the receptor is sensitized by an agonist, during which the channel opens resulting in increasing inwardly cationic currents following ATP exposure which slowly tends towards a plateau. This leveling off of the current is reffered to as desensitisation and following this the current amplitude rapidly goes to zero when the ATP becomes unbound. P2X_4_ and P2X_7_ receptors differ in terms of the kinetic machinery actively involved in their sensitivity and function.

In the model, ATP application is potentiated though two forward rate constants of *α*_1_ and *α*_2_. All backward rates were chosen as variable functions. Experimental data show that desensitisation of P2XRs depends on the level of ATP exposure [23,29,33,34]. Therefore, the gating transition between S⇄D has an exponential dependency on the agonist. This prevents the desensitisation pathway from disappearing in the model during agonist removal since the exponential terms became unity upon ATP removal. Note that both exponential rates are present in both sides of the transitions guaranteeing the amount of reduction in S state in the forward reaction returns to D state in a proportionally balanced fashion. The model conserves all the species because it is a closed system; therefore the the sum of all state variables at any time is equal to unity and the system state variables return to their initial resting state after stimulation has ceased. An important function of a biophysical model is that the state of the system should go back to its resting state, because the model can be activated again without affecting the model predictions, for example, when this model is simulated using a repeated application of ATP. In order to ensure this property, the transition between D and C states through a negative exponential rate function makes an extra path that enables D to be depleted to C when O becomes very small (note that the path is activated because the exponential function goes to its high value when O significantly decreases).

**Fig 3:**
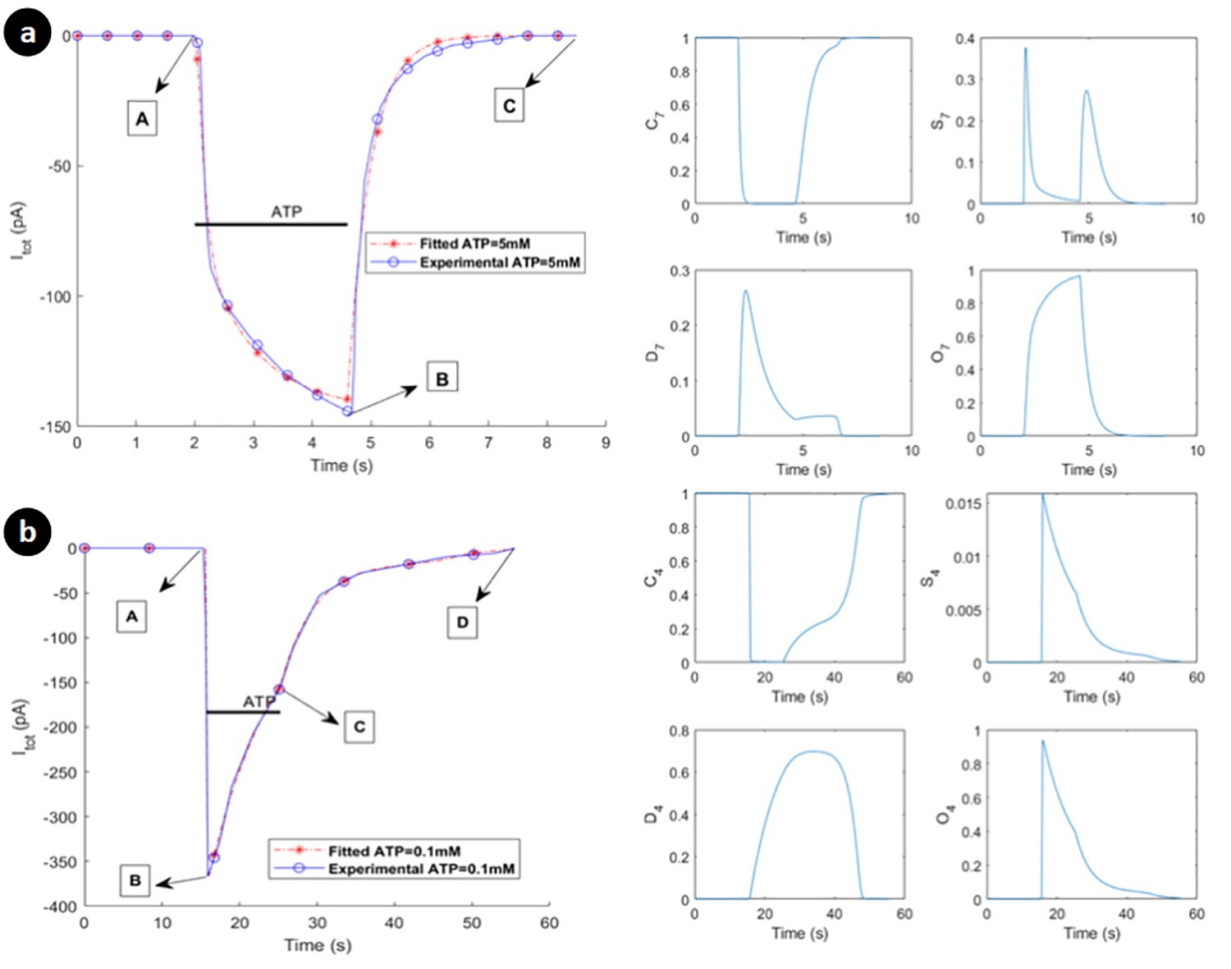
Current transient (left panel) and receptor gate C/S/D/O state responses (right panel) upon ATP treatment of (a) 5mM ATP within 2.6 seconds via activation hP2X_7_ receptor, (b) and 0.1mM ATP within 9.6 seconds via activation hP2X_4_ in microglia. Horizontal bars show the duration of agonist exposures. Experimental data come from [22,23]. Corresponding states for both receptors on right panels have different transient behavioros that are mainly due to the distiction between model parameters and model structure.

The microglial hP2X_7_ receptor currents [22] have two significant phases: A to B (activation/sensitisation/desensitisation) and B to C (deactivation). The exponential backward rates in Fig 2 between O/S and S/C allows this complex behaviour to be captured. The function *φ* for the hP2X_7_ model is defined as *φ*(A) = A. The exponential backward rates in Fig 2 between O/S and S/C makes this complex behaviour possible to be captured in such a simple manner. In fact, when agonist is high the exponential terms are low and let the model pass the flow in the forward direction (C/S/O). By contrast, these two terms switch to their maximal values (viz. one) upon agonist removal, so letting the state O deplete (go to zero) in order to capture the deactivation phase (B to C) in Fig 3A. Strictly speaking, such intricate dynamics can be captured by simpler models when reaction rates are desiged such that they dynamically change to avail of existing paths of the model. They are responsible for reproducing the main properties of the experimental data for whole-cell current, such as persistent sensitised state and monophasic/biphasic dynamics.

In contrast, hP2X_4_ receptors mediate a rapid current that is sustainably greater than the peak amplitude of P2X_7_ receptor as shown in Fig 3B, and their desensitisation is much faster than P2X_7_R, which is also validated by the model predictions. As seen in Fig 3B, hP2X_4_ receptor has three significant phases at macroscopic level: A to B (activation/sensitisation), B to C (desensitisation), and C to D (deactivation). In contrast to microglial rP2X4 [24], the hP2X_4_ current takes some time to vanish after ATP removal (C to D). This additional phase requires minor changes to the P2X model of Fig 2, where $\varphi (A)=e^{\zeta_{1}A}$ is chosen. The transition between S and D states are gated by an exponential form of the agonist in Fig 2. It allows the model does not need additional state variables and therefore avoids the necessity for a more complex model. When ATP is zero, the transition between C and S is eliminated while *φ*(A) becomes unity. In fact, the bidirectional path between S/D and S/O makes active routes for the current to vanish, namely, the phase of C to D in Fig 3B is simply realised. S5 Text explains the relevance and necessity of model elements in Fig 2 with more quantitative details.

To derive the mathematical equations representing the kinetic P2X model, the topology in Fig 2 is now translated into a system of time-dependent coupled ordinary differential equations as expressed in Eqs (1–4) by using the law of mass action:

$$\frac{dC_{Z}}{dt}=\beta_{1Z}e^{{-\zeta }_{1Z}A}S_{Z}+\beta_{4Z}e^{{-\zeta }_{2Z}O_{Z}}D_{Z}-\alpha_{1Z}AC_{Z}$$ (1)

$$\frac{dS_{Z}}{dt}=\alpha_{1Z}AC_{Z}+\beta_{2Z}e^{{-\zeta }_{1Z}A}O_{Z}+\beta_{3Z}e^{\zeta_{1Z}A}D_{z}-(\beta_{1Z}e^{{-\zeta }_{1Z}A}+\alpha_{2Z}\varphi_{Z}(A)+\alpha_{3Z}e^{\zeta_{1Z}A})S_{Z}$$ (2)

$$\frac{dD_{Z}}{dt}=\alpha_{3Z}e^{\zeta_{1Z}A}S_{Z}-(\beta_{3Z}e^{\zeta_{2Z}A}+\beta_{4Z}e^{{-\zeta }_{2Z}O_{Z}})D_{Z}$$ (3)

$$\frac{dO_{Z}}{dt}=\alpha_{2Z}\varphi_{z}(A)S_{Z}-\beta_{2Z}e^{{-\zeta }_{1Z}A}O_{Z}$$ (4)

Each state C, S, D and O corresponds to the fraction of a specific P2X receptor that are in the *Closed*, *Sensitised*, *Desensitised* and *Opened* state at any given time. The above equations are used for both P2X models (P2X_4_ and P2X_7_), where *Z* is used to denote the subtype receptor number (4 or 7). The fitting of the model parameters is discussed in S1 Text. Values and units of rate constants and coefficients in Eqs (1–4) are provided in the *model fitting and validation* section.

### hP2X_Z_ current model

Having the system of Eqs (1) to (4), we can now derive the current equations for the P2XRs (4 and 7). Currents through the receptors are proportional to the state O_Z_, where Z is the subchannel number of the receptor, and can be expressed as terms of the familiar conductance channel model. These currents are obtained by a product of the open state, the maximum conductance *g* through the channels, and the difference between the membrane potential (*V*_*m*_) and the reversal potential of the cell (*E*). The Ca^2+^ and Na^+^ currents for the receptors can be modelled as two separate conductance channels using Eqs (5) and (6), as illustrated in Fig 1B, where reversal potentials are expressed in Eqs (8) and (9). With regards to K^+^ efflux, the mechanisms underpinning this ionic current are poorly understood and are therefore not considered in this paper. However, the influx of Na^+^ will affect the NCX and will consequently have a downstream effect on Ca^2+^. Therefore, the contribution to the Na^+^ concentration in the cytoplasm by the P2XRs needs to be considered for a more complete understanding of the observed Na^+^ and Ca^2+^ dynamics. This approach, where a conductance type model is used to characterise the P2XRs as individually ATP-gated Ca^2+^ and Na^+^ channels, allows the segregation of the Ca^2+^ or Na^+^ channel currents while maintaining a similar overall time-dependant response. While we recognise that this is an assumption, the segregation of these two ions facilitate the calculation of both Ca^2+^ or Na^+^ ionic concentrations in the cytoplasm. Ca^2+^ or Na^+^ channel conductance in the proposed model is set to define channel stoichiometry. The total current is calculated by the summation of the separate channel contributions, as shown in Eq (7).


$$I_{{hP2XZ}_{Na}}=g_{{hP2XZ}_{Na}}O_{Z}(V_{m}-E_{Na})$$ (5)

$$I_{{hP2XZ}_{Ca}}=g_{{hP2XZ}_{Ca}}O_{Z}(V_{m}-E_{Ca})$$ (6)

$$I_{{tot}_{hP2Z7}}=I_{{hP2XZ}_{Na}}+I_{{hP2XZ}_{Ca}}$$ (7)

The above equations are used for both P2X models (P2X_4_ and P2X_7_), where *Z* is used to denote the subtype receptor number (4 or 7). The ionic concentrations in the extracellular space are assumed constant but are dynamic in the cytoplasm due to the influx/efflux of ions giving rise to a dynamic transmembrane concentration gradient. Accordingly, the reversal potentials for Na^+^ and Ca^2+^ in Eqs (5) and (6) can be expressed in terms of the Nernst equation as

$$E_{\mathrm{Na}}=\frac{RT}{Z_{{\mathrm{Na}}^{+}}\times F}ln\left(\frac{\left[{Na}_{x}^{+}\right]}{\left[{Na}_{i}^{+}\right]}\right)$$ (8)

$$E_{Ca}=\frac{RT}{Z_{{\mathrm{Ca}}^{2+}}\times F}ln\left(\frac{\left[{Ca}_{x}^{2+}\right]}{\left[{Ca}_{i}^{2+}\right]}\right)$$ (9)

where *x* and *i* are are the extracellular and intracellular space respectively, $Z_{{\mathrm{Na}}^{+}}$ and $Z_{{\mathrm{Ca}}^{2+}}$ are the valencies of Na^+^ and Ca^2+^ respectiveley, *F* is Faradays constant, *R* is Boltzmann constant and *T* is temperature in kelvins.

### Sodium calcium exchanger (NCX)

The NCX is an antiporter that electrochemically exchanges three Na^+^ ions for one Ca^2+^ ion across the cell membrane [35]. This exchanger works in two different operational modes (forward or reversed) depending on *V*_*m*_ and the transmembrane Na^+^ and Ca^2+^ gradients. The Na^+^ and Ca^2+^ current densities of the exchanger are defined by

$$J_{NaNCX}={\bar{J}}_{NCX}{\left(\frac{\left[{Na}_{i}^{+}\right]}{\left[{Na}_{x}^{+}\right]}\right)}^{3}e^{\frac{\gamma FV_{m}}{RT}}-\frac{\left[{Ca}_{i}^{2+}\right]}{\left[{Ca}_{x}^{2+}\right]}e^{\frac{(\gamma -1)FV_{m}}{RT}}$$ (10)

$$J_{CaNCX}=-\frac{2}{3}J_{NaNCX}$$ (11)

where ${\bar{J}}_{NCX}$ and *γ* are the NCX conductance and partition number, respectively [35]. The current density *J*_*NaNCX*_ has been obtained through a membrane-coupled protein that assumes both Na^+^ and Ca^2+^ are abundant in the extracellular space. The current density therefore depends on the intracellular and extracellular concentrations of ions and *V*_*m*_. The exchanger has a low affinity and binds moderately to Ca^2+^. It transmits both Na^+^ and Ca^2+^ ions quickly because of its high capacity and consequently the NCX is mainly responsible for removing Ca^2+^ that flows into the cytosol.

### Plasma membrane Ca^2+^ ATPase (PMCA)

The PMCA pump is a high-affinity protein that preserves low cytosolic Ca^2+^ profiles by consuming ATP macromolecules and extrudes Ca^2+^ from inside the microglia against its ionic gradient. The Ca^2+^ current density through PMCA is modelled using Michaelis-Menten kinetics [36], and is formulated by

$$J_{PMCA}={\bar{J}}_{PMCA}\frac{{\left[{Ca}_{i}^{2+}\right]}^{n}}{{\left[{Ca}_{i}^{2+}\right]}^{n}+{K_{PMCA}}^{n}}$$ (12)

where ${\bar{J}}_{PMCA}$ and *K*_*PMCA*_ are the maximum current density and pump affinity respectively. *n* is the order of Hill function, which is set to one for this study. This pump removes Ca^2+^ and is also used to maintain Ca^2+^ at low levels in our model.

### Na^+^ and Ca^2+^ leakage channels

In our model, two separate membrane bound leakage channels exist for Na^+^ and Ca^2+^ and are modelled as two passive electrochemical gradients by assuming a Nernst-like equation given by

$$J_{NaLeak}=g_{NaLeak}(V_{m}-E_{Na})$$ (13)

$$J_{CaLeak}=g_{CaLeak}(V_{m}-E_{Ca})$$ (14)

where *g*_*y*_ and *E*_*y*_ are a conductance and a reversal potential for ion *y*, and *V*_*m*_ is the membrane potential. The physiological conditions of these channels change dynamically with both membrane and reversal potentials.

### Na^+^ and Ca^2+^ concentration dynamics

Na^+^ and Ca^2+^ signalling is formulated as a fluid compartment model, which is assumed to be well mixed. The changes in $\left[{\mathrm{Na}}_{i}^{+}\right]$ and $\left[{\mathrm{Ca}}_{i}^{2+}\right]$ are modelled as

$$\frac{d[{\mathrm{Na}}_{i}^{+}]}{dt}=-\frac{\frac{I_{{hP2X7}_{Na}}}{S_{m}}+\frac{I_{{hP2X4}_{Na}}}{S_{m}}+J_{NaNCX}+J_{NaLeak}}{Z_{{\mathrm{Na}}^{+}}\times F\times V_{p}}\times S_{p}$$ (15)

$$\frac{d[{\mathrm{Ca}}_{i}^{2+}]}{dt}=-\frac{\frac{I_{{hP2X7}_{Ca}}}{S_{m}}+\frac{I_{{hP2X4}_{Ca}}}{S_{m}}+J_{CaNCX}+J_{PMCA}+J_{CaLeak}}{Z_{{\mathrm{Ca}}^{2+}}\times F\times V_{p}}\times S_{p}$$ (16)

where *S*_*m*_, *S*_*p*_, *Z*, *F* and *V*_*p*_ are the microglial membrane surface area, microglial process surface area, the valency of ions, Faradays constant and the intracellular process volume respectively: the model assumes that $\left[{\mathrm{Na}}_{i}^{+}\right]$ and $\left[{\mathrm{Ca}}_{i}^{2+}\right]$ reside in the total cytosolic volume of microglial processes. The conversion of the P2XR’s whole-cell patch-clamp currents into current densities is performed by dividing over *S*_*m*_ due to other current components already being expressed as current densities.

### Membrane potential

Taking the same approach as is used for neuronal cells [32,37], *V*_*m*_ can be related to ionic flow across the membrane as

$$\frac{dV_{m}}{dt}=-\frac{1}{C_{m}}\times [I_{{tot}_{hP2X7}}+I_{{tot}_{hP2X4}}+(J_{NaNCX}+J_{CaNCX}+J_{PMCA}+J_{NaLeak}+J_{CaLeak})\times S_{p}]$$ (17)

where *C*_*m*_ is the membrane capacitance. The total current flow through P2X channels are added to the transmembrane currents across NCX exchanger, PMCA pump and leak channels. The terms expressed as current densities in Eq (17) are multiplied by *S*_*p*_.

### Model fitting and validation

In this section, we fit (see S1 Text) and validate our P2X models using available experimental data [22,23]. As illustrated in Fig 3, the kinetic P2X model shown in Fig 2 is able to reproduce all the major gating properties of hP2X_7_ and hP2X_4_ receptors for the whole-cell current experiments, i.e. activation, deactivation, sensitisation and desensitisation. The fitted hP2X_7_ model is subject to 5mM ATP stimulation [22], while 0.1mM ATP had been used for the hP2X_4_ receptor [23].

In contrast to hP2X_4_ current profile that peaks at -372pA, hP2X_7_ responds much slower reaching a peak of -145pA. Clearly, both models closely approximate the experimental data. The right hand side of Fig 3 illustrates transient behaviour of the whole-cell current state variables. As seen, sensitisation and desensitisation significantly differ in both models. Interestingly, there are two similar transients in state S of Fig 3A that align with the the existing two phases of activation and deactivation. The first transient relates to the activation phase from point A to B (Fig 3A) where ATP is present, and the second transient is created upon ATP removal since the state O must be depleted from the only available path (O to S, followed by S to C or S to D and then finally to C in Fig 2A). The summation of transient responses of all the state variables are equal to unity at all times and all state variables return to their resting state post stimulation. There are four classes of parameters in the entire model formulated by Eqs (1) to (17), including, (a) whole-cell patch-clamp current parameters of α and β, (b) estimated constant parameters such as ζ, (c) morphological parameters such as *C*_*m*_ and *S*_*p*_, and (d) physically measured parameters such as *K*_*PMCA*_ and *γ* used from the literature. Conductances of both P2X models were fitted to clamped electrophysiological voltage data [22,23] and then reduced by a factor of 25 (similar to [30]) such that the difference between the resting [Ca^2+^] and the peak [Ca^2+^] (as can been seen later inresults section) lies between 50nM and 55nM, in agreement with the general P2X-mediated $\left[{\mathrm{Ca}}_{i}^{2+}\right]$ dynamics found in the literature [38]. PMCA, NCX and leak channel parameters were determined through numerical simulation to fit experimental data [39] such that $\left[{\mathrm{Na}}_{i}^{+}\right]$ and $\left[{\mathrm{Ca}}_{i}^{2+}\right]$ are maintained at their resting concentrations in steady state. This is a paramount property of the microglial cells where the model takes up the role of maintaining $\left[{\mathrm{Ca}}_{i}^{2+}\right]$ in resting microglia because microglia must be sufficiently sensitive to their microenvironment to generate a quick response to injury. By letting the optimiser calibrate the value of *ζ* parameters in Fig 2, it was unable to give a satisfactory fit quality. Hence, *ζ* was estimated manually by setting different values for *ζ* and letting the optimiser find all remaining parameters. Therefore, trial and error was used where values of *ζ*_1_ and *ζ*_2_ greater than 1 was chosen, and these values were then used in the optimiser. This process was repeated until the best fit for all remianing parameters was found (see Table 1 for final values of *ζ*_1_ and *ζ*_2_). S5 Text gives an example for choosing a typical value of *ζ*_1_ in that the model cannot capture the experimental data well.

Morphological changes occur in microglia and while the proposed model does not take this into account, morphological data [40] was used to estimate the surface area and volume of activated microglia by assuming three cylindrical processes and the cell body is represented by a cube: while microglia can dynamically lose their spherical somatic phenotype upon activation, a cube is closer to an image of particular microglia morphology as reported in [40]. Using these assumptions, the process surface area and volume can be calculated and used as parameters in Eqs (15) to (17). Values, units and descriptions of the parameters used in our model are presented in Table 1.

**Table 1:** Parameters for the mathematical model of P2X-mediated calcium and sodium dynamics in human microglia.

Parameter	Value	Unit	Description	Source
***α***_**17**_	2427.508	M^-1^s^-1^	Rate constant for C_7_→S_7_	Fitted
***α***_**27**_	1473.074	M^-1^s^-1^	Rate constant for S_7_→O_7_	Fitted
***α***_**37**_	0.0299	s^-1^	Rate constant for S_7_→D_7_	Fitted
***β***_**17**_	4.277	s^-1^	Rate constant for *S*_7_→C_7_	Fitted
***β***_**27**_	2.616	s^-1^	Rate constant for O_7_→S_7_	Fitted
***β***_**37**_	0.0125	s^-1^	Rate constant for O_7_→S_7_	Fitted
***β***_**47**_	930.251	s^-1^	Rate constant for D_7_→C_7_	Fitted
***α***_**14**_	187057.5	M^-1^s^-1^	Rate constant for C_4_→S_4_	Fitted
***α***_**24**_	1111.369	s^-1^	Rate constant for S_4_→O_4_	Fitted
***α***_**34**_	6.223	s^-1^	Rate constant for S_4_→D_4_	Fitted
***β***_**14**_	7.298	s^-1^	Rate constant for S_4_→C_4_	Fitted
***β***_**24**_	19.369	s^-1^	Rate constant for O_4_→S_4_	Fitted
***β***_**34**_	0.01341	s^-1^	Rate constant for D_4_→S_4_	Fitted
***β***_**44**_	528.077	s^-1^	Rate constant for D_4_→C_4_	Fitted
***ζ***_**17**_	1×10^3^	M^-1^	P2X_7_ exponential rate functions coefficient	Estimated
***ζ***_**27**_	1×10^3^		P2X_7_ exponential rate functions coefficient	Estimated
***ζ***_**14**_	0.2×10^3^	M^-1^	P2X_4_ exponential rate functions coefficient	Estimated
***ζ***_**24**_	0.2×10^3^		P2X_4_ exponential rate functions coefficient	Estimated
$g_{{hP2X7}_{Na}}$	10.33×10^−12^	S	Maximal hP2X_7_ sodium conductance	Fitted
$g_{{hP2X7}_{Ca}}$	6.91×10^−12^	S	Maximal hP2X_7_ calcium conductance	Fitted
$g_{{hP2X4}_{Na}}$	31.28×10^−12^	S	Maximal hP2X_4_ sodium conductance	Fitted
$g_{{hP2X4}_{Ca}}$	20.85×10^−12^	S	Maximal hP2X_4_ calcium conductance	Fitted
***C***_**7**_**|**_**0**_ **= *C***_**4**_**|**_**0**_	1		Initial values of C_7_ and C_4_	
***S***_**7**_**|**_**0**_ **= *S***_**4**_**|**_**0**_	0		Initial values of S_7_ and S_4_	
***D***_**7**_**|**_**0**_ **= D**_**4**_**|**_**0**_	0		Initial values of D_7_ and D_4_	
***O***_**7**_**|**_**0**_ **= *O***_**4**_**|**_**0**_	0		Initial values of O_7_ and O_4_	
${\bar{J}}_{NCX}$	55	Am^-2^	Maximum NCX current density	Estimated
***γ***	0.5		NCX partition number	[35]
***F***	96485	CMol^-1^	Faraday constant	[35]
***R***	8.314	JMol^-1^K^-1^	Ideal gas constant	[35]
*T*	310	K	Temperature	[35]
${\bar{J}}_{PMCA}$	0.06	Am^-2^	Maximum PMCA current density	Estimated
***K***_***PMCA***_	0.1×10^−6^	M	PMCA Ca^2+^ affinity	[36]
$Z_{{\mathrm{Na}}^{+}}$	1		Na^+^ valency	[35]
***g***_***NaLeak***_	305×10^−4^	Sm^-2^	Na^+^ leak conductane	Calculated
$Z_{{\mathrm{Ca}}^{2+}}$	2		Ca^2+^ valency	[35]
***g***_***CaLeak***_	783×10^−4^	Sm^-2^	Ca^2+^ leak conductance	Calculated
${\left[{Na}_{i}^{+}\right]}_{0}$	8×10^−3^	M	Initial ${Na}_{i}^{+}$ concentration	[35]
${\left[{Ca}_{i}^{2+}\right]}_{0}$	45×10^−9^	M	Initial ${Ca}_{i}^{2+}$ concentration	[35]
$\left[{Na}_{x}^{+}\right]$	130×10^−3^	M	Extracellular Na^+^ concentration	[35]
$\left[{Ca}_{x}^{2+}\right]$	2×10^−3^	M	Extracellular Ca^+^ concentration	[35]
***V***_***m***_ **|**_**0**_	-0.06	V	Initial membrane potential	[22]
***C***_***m***_	12×10^−12^	F	Microglia membrane capacitance	[30]
***S***_***m***_	3.178×10^−10^	m^2^	Microglia membrane surface area	[40]
***S***_***p***_	2.435×10^−10^	m^2^	Microglia process surface area	[40]
***V***_***p***_	1.67×10^−13^	L	Microglia process volume	[40]

## Results

The model developed in the previous section is now studied by numerical simulation to provide a deeper insight into intracellular P2X-triggered Na^+^ and Ca^2+^ dynamics in microglial cells. The model was implemented in MATLAB Release 2021a. We used *ode23s* routine to numerically integrate the non-linear, stiff model equations. The integration was carried out using the default MATLAB ODE solver timestep because it takes advantage of dynamic step sizes for implicit numerical integration. For integration stability during the curve fitting and numerical simulation, the calculation of the right-hand-side terms of the ODEs in Eqs (1) through (17) and the Jacobian matrix of the system equations were implemented for the model. This section investigates four case studies, including, whole-cell current due to P2XRs, Ca^2+^, Na^+^ and V_m_ dynamics resulting from the P2X-provoked currents.

### P2X-mediated whole-cell currents

**Fig 4:**
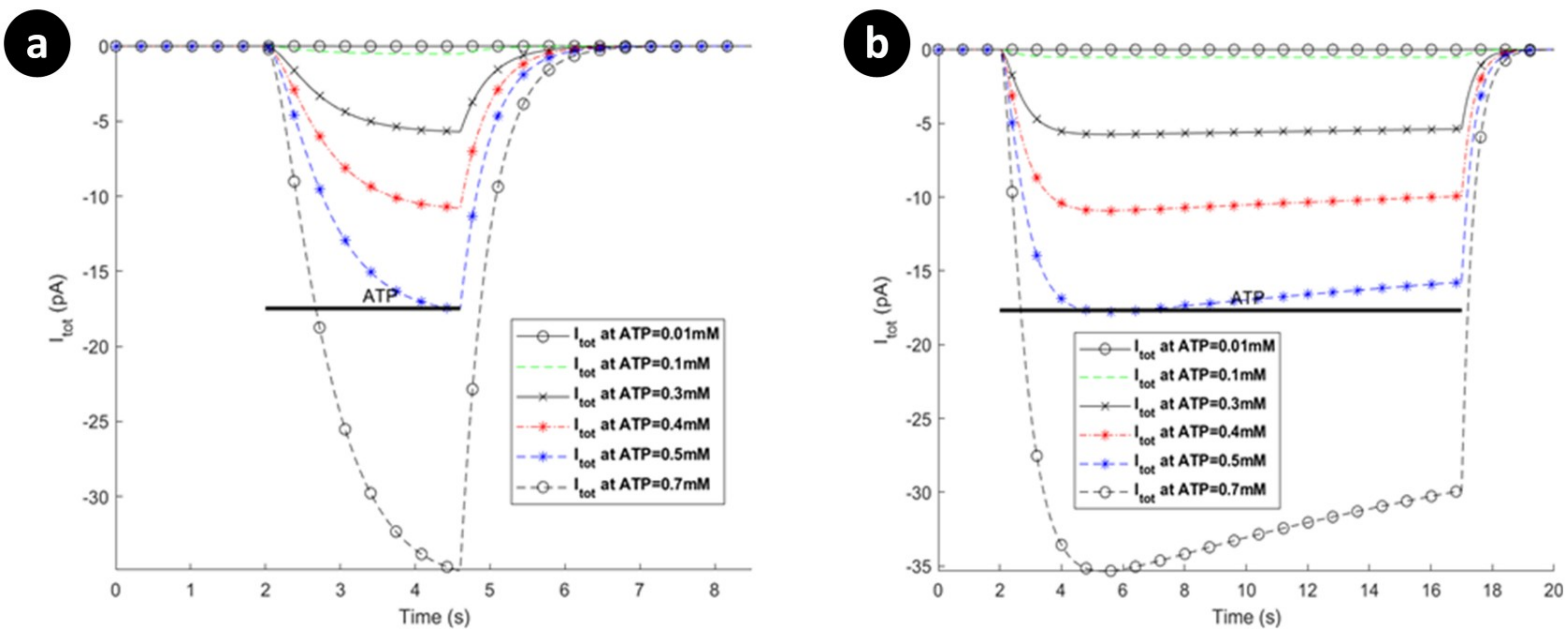
Dependence of total P2X_7_ currents on different ATP treatment under 1 millimolar. (a) 3 second application, and (b) 15 seconds application via activation of P2X_7_ for human microglia. Horizontal bars show duration of agonist exposures.

**Fig 5:**
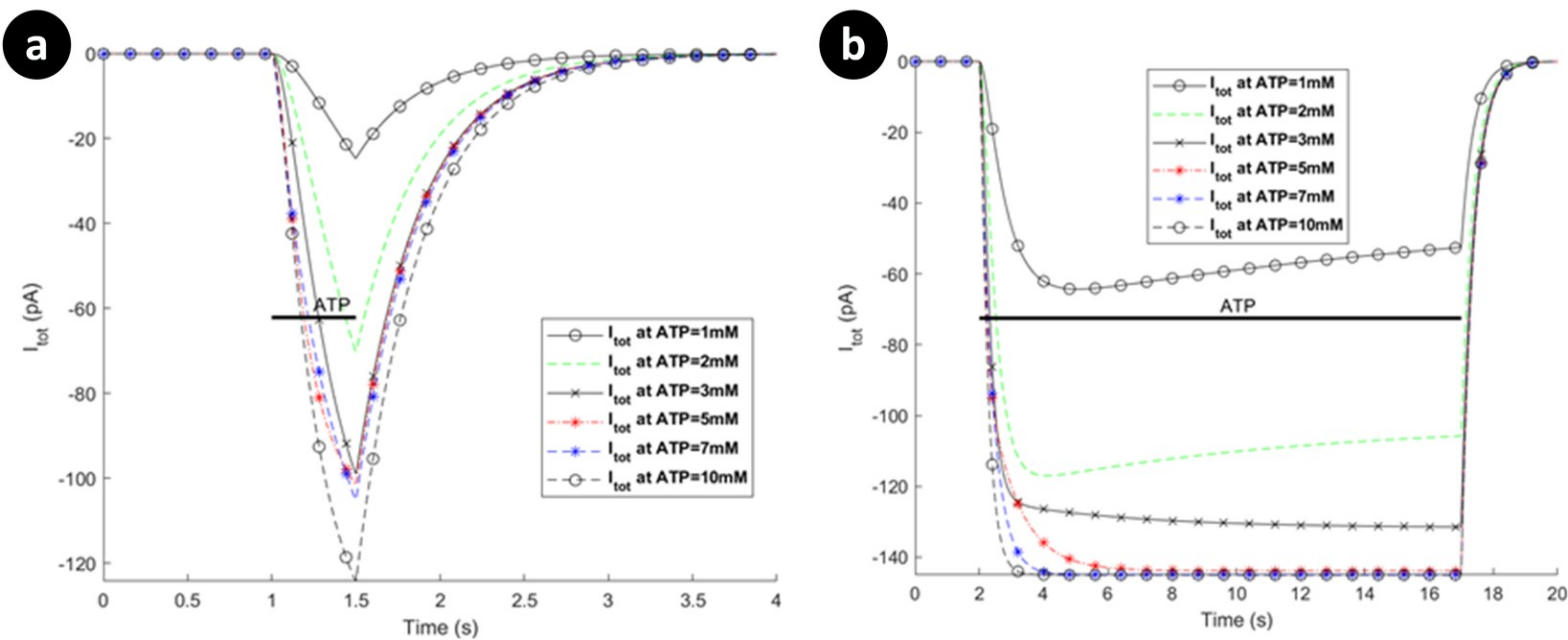
Total P2X_7_ currents on different ATP treatments above 1 millimolar for human microglia: (a) short duration of ATP application (0.5 seconds), versus (b) long duration of ATP exposure (15 seconds).

**Fig 6:**
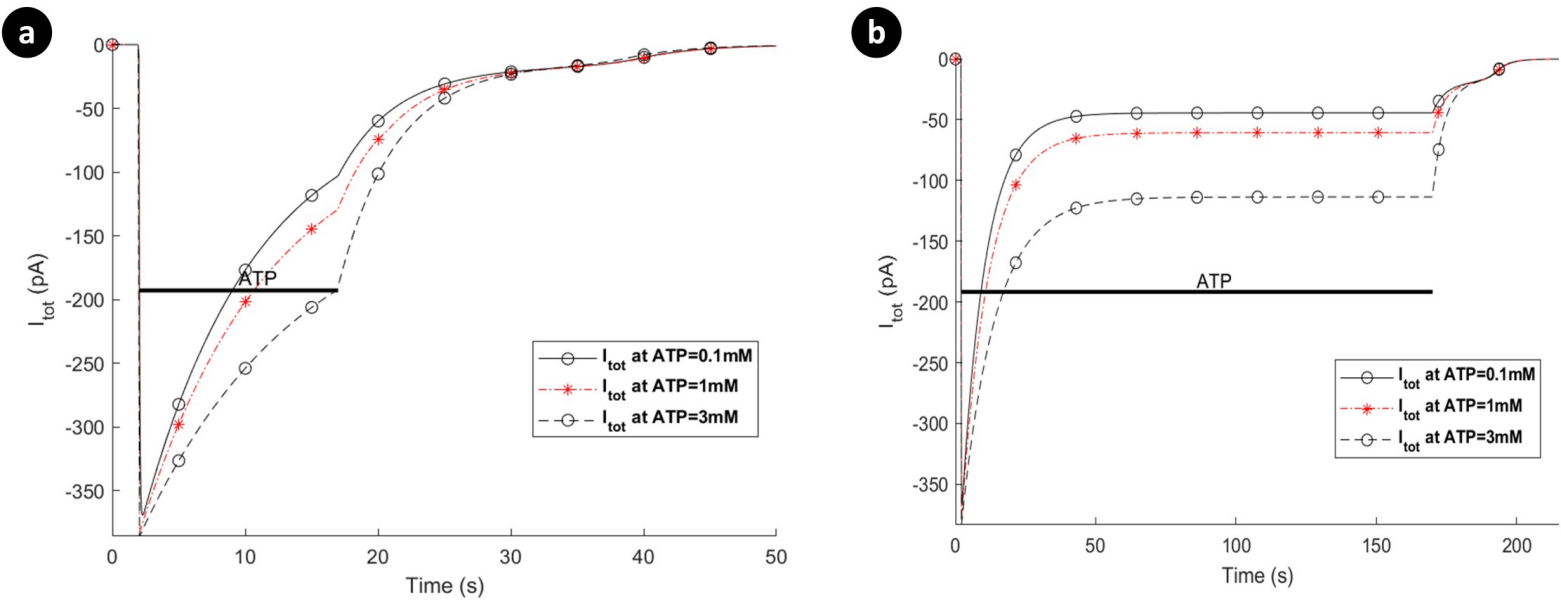
Prediction of patterns in total currents via activation of P2X_4_ for human microglia. The curves represent biphasic responses: (a) short duration of ATP exposure, versus (b) long duration of ATP treatment.

Microglial hP2X_4_ and hP2X_7_ receptors are activated in micromolar and millimolar concentrations of the applied ATP, respectively [24,38]. We now consider model predictions for both receptors. The simulated whole-cell current responses of hP2X_7_ and hP2X_4_ receptors are shown in Figs 4–6 for different applications of ATP stimulus level and duration.

All the results for hP2X_7_ receptor invoked by micromolar or millimolar ATP (see Figs 4 and 5 respectively) display two biphasically distinguishable phases as observed in [22], namely, current initialisation (rapid effect of ATP) and time-dependent current facilitation. Fig 5A illustrates a short ATP stimulus in contrast to Fig 5B where, at 1mM and 2mM, the behaviour conforms to the Ca^2+^ dynamics reported experimentally [22]. Note that: it is inferred that ATP levels of 1-2mM corresponds to 300μM of Benzoylbenzoyl-ATP (Bz-ATP) [41]. This is also discussed further in the next section.

The predictions in Fig 5A and 5B show that for short ATP application, the current remains in ascendancy while for long ATP stimulus the current plateaus and thereafter slowly declines. In contrast to rat microglial data [42], the current amplitude appears to saturate with larger ATP levels, which suggests that desensitisation becomes more pronounced for higher levels of ATP. However, this behaviour agrees with what has been reported for mouse P2X_7_ microglia receptors [43].

The predictions in Fig 6A show that the amplitude and rate of increase of current is largely unaffected by the magnitude of the ATP stimulus for hP2X_4_R. Note that this receptor appears to rapidly desensitise until the ATP stimulus is removed, thereafter the current falls off rapidly to baseline. In contrast, Fig 6B shows that for long ATP durations the current reaches a steady state after a period of desensitisation and these levels are ATP dependant. Only when the ATP stimulus is removed does the current return to its baseline level. This dual component indicates a biophsyical fingerprint that has been reported through *in vitro* electrophysiology [44]. For example, human monocyte-derived macrophage research shows that P2X_4_Rs exhibit fast activation current followed by biphasic desensitisation in [34], particularly for high levels of ATP. Pertinent to dynamic microglia, this biphasic fucntionality of P2X_4_Rs may provide a regulatory pathway to control motility as has been suggested in expression systems [27].

The hP2X_7_ and hP2X_4_ receptor models can now be integrated yielding valuable data regarding P2X-mediated Ca^2+^ and Na^+^ signalling in human microglia. These data could be used to guide future experimental design.

### P2X-mediated calcium dynamics

**Fig 7:**
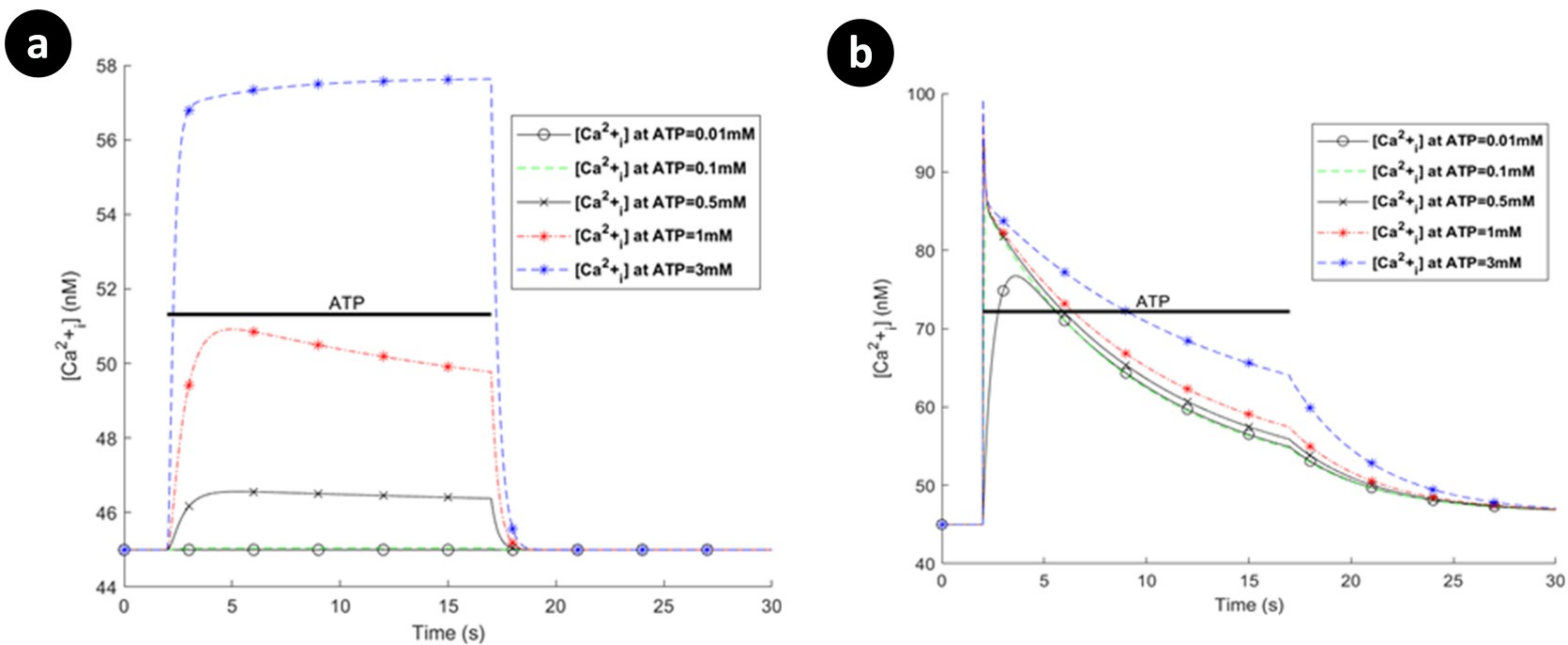
Intracellular $\left[{Ca}_{i}^{2+}\right]$ transients when (a) the hP2X_4_ receptor is switched off with hP2X_7_ active, and (b) hP2X_7_ is switched off with hP2X_4_ active.

The interaction between the P2XRs, NCX, PMCA and leak channels dictates the dynamics of Ca^2+^ and Na^+^ concentrations and also *V*_*m*_, as described by Eqs (15) to (17). The model predicts the total Ca^2+^ influx/efflux via the receptors, leak channels, extruders and pumps. Fig 7 shows individual Ca^2+^ transients for each receptor separately assuming the same ATP exposure level and duration. For Fig 7A, the $\left[{Ca}_{i}^{2+}\right]$ reaches a plateau for higher levels of ATP and this correlates with the total current carried by this receptor (e.g., at 3mM), as shown in Fig 5B. Fig 7B agrees with experimental intracellular Ca^2+^ measurements [22] for ATP = 1mM, as discussed earlier, in which there exists a local maximum before the Ca^2+^ curve goes to its plateau prior to ATP removal. Fig 7B shows a very transient-like $\left[{Ca}_{i}^{2+}\right]$ profile where hP2X_4_R preserves $\left[{Ca}_{i}^{2+}\right]$ for higher levels of ATP which is consistent with its equivalent current profile in Fig 6.

**Fig 8:**
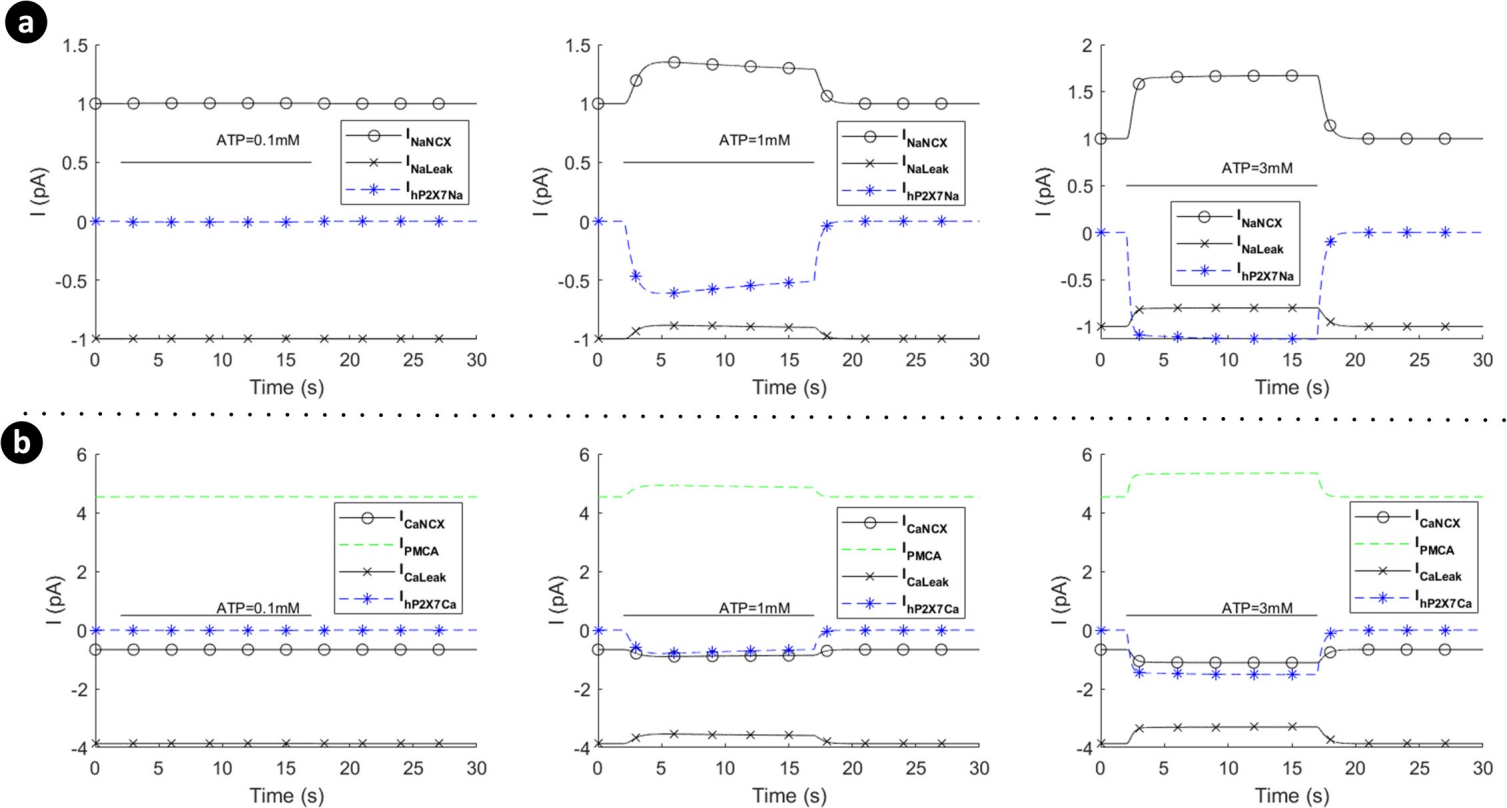
Dynamics of absolute currents via activation of hP2X_7_ when the hP2X_4_ receptor is switched off. Top (a) and bottom (b) panels respectively quantify the sodium (a) and calcium components of the model receptor for 0.1mM, 1mM and 3mM ATP.

Fig 8 shows the absolute currents carried by the P2X_7_ (P2X_4_ switched off), NCX, PMCA and leak channels for ATP levels of 0.1mM, 1mM and 3mM. Note that prior to the onset of the ATP stimulus Ca^2+^ efflux by the PMCA is negated by the NCX operating in reverse mode and calcium leak across the membrane via the Ca^2+^ leak channel. As the ATP stimulus is applied, the P2X_7_ receptor is activated and both Ca^2+^ and Na^+^ current flows into the cytoplasm. However, *V*_*m*_ and $\left[{Ca}_{i}^{2+}\right]$ are the primary drivers for the NCX ensuring that it operates in reverse mode, as can be seen in Fig 8 for higher levels of ATP (1mM and 3mM). Also, after the initial disturbance, both the Ca^2+^ and Na^+^ currents stabilise such that the net Ca^2+^ or Na^+^ current flowing across the membrane becomes zero, and the concentration of both ions in the cytoplasm stabilises, in agreement with Fig 7A.

**Fig 9:**
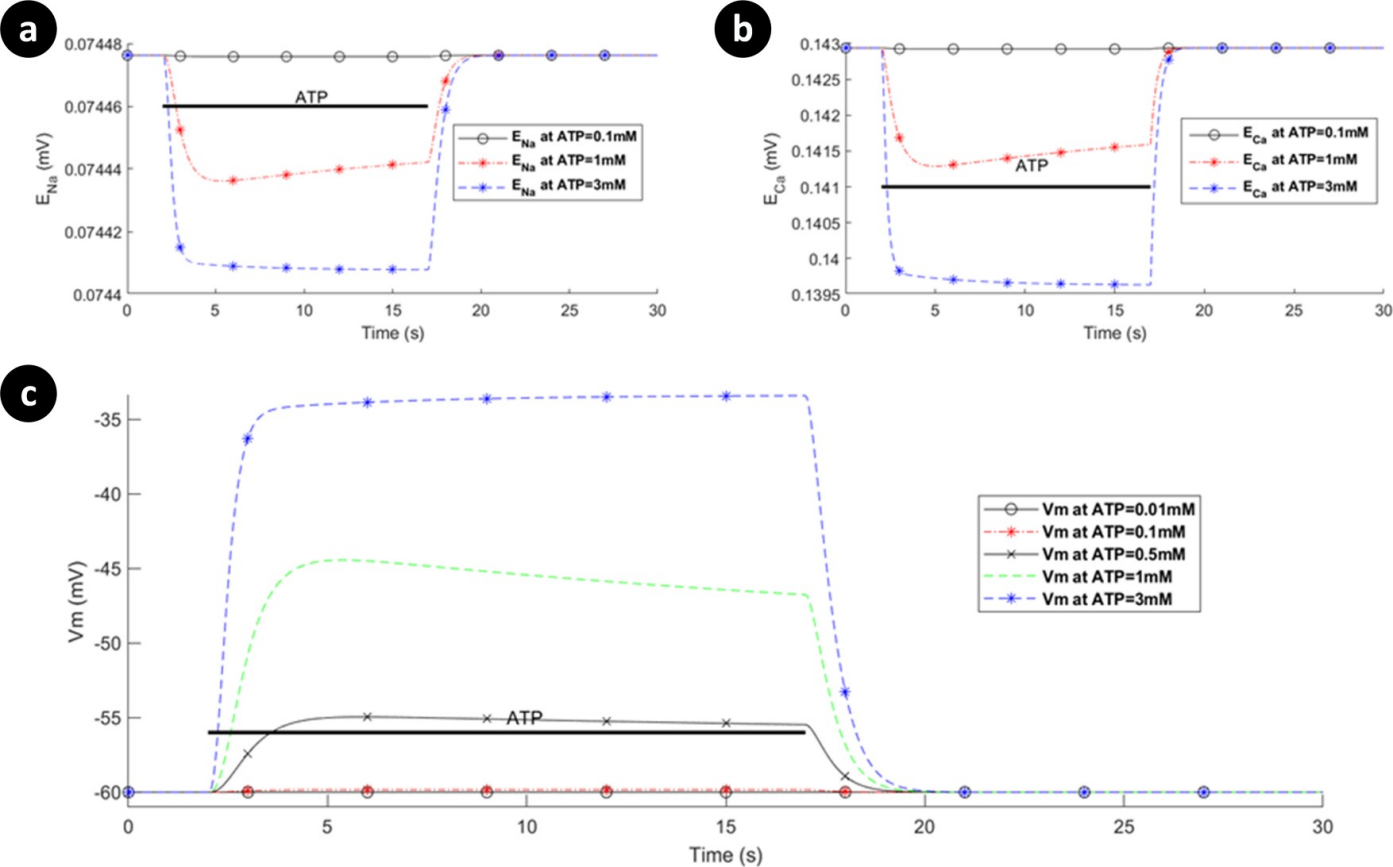
Dynamics of the reversal potentials associated with (a) Ca^2+^, (b) Na^+^, and (c) the membrane potential as a function of ATP levels when the hP2X_4_ receptor is turned off.

Reversal potentials and *V*_*m*_ for P2X_7_R are shown in Fig 9 when P2X_4_R is switched off. Note that the reversal potential for Na^+^ (Fig 9) changes much less significantly in comparison with $\left[{Ca}_{i}^{2+}\right]$, because the baseline level of $\left[{Na}_{i}^{+}\right]$ is much higher than that of $\left[{Ca}_{i}^{2+}\right]$. Additionally, experimental data [42] indicates that rectification of *V*_*m*_ occurs following low levels of ATP stimulus while sustained depolarisation occurs with higher ATP concentrations. Our model of *V*_*m*_ (illustrated in Fig 9C) shows a similar trend to this data.

**Fig 10:**
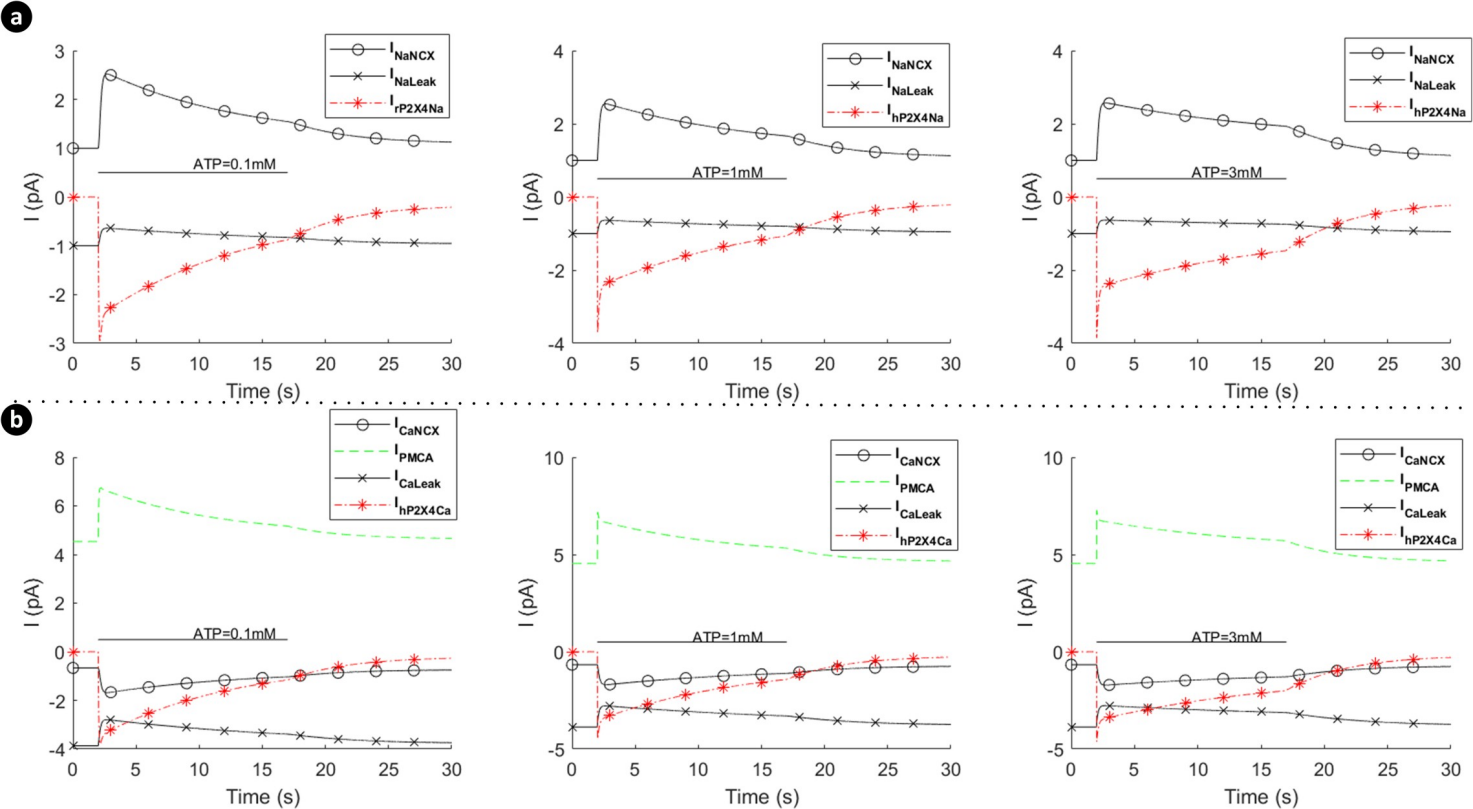
Dynamics of absolute currents via activation of hP2X_4_ receptor when the hP2X_7_ receptor is turned off. Top (a) and bottom (b) panels respectively quantify the sodium and calcium components of the model for 0.1mM, 1mM and 3mM ATP.

**Fig 11:**
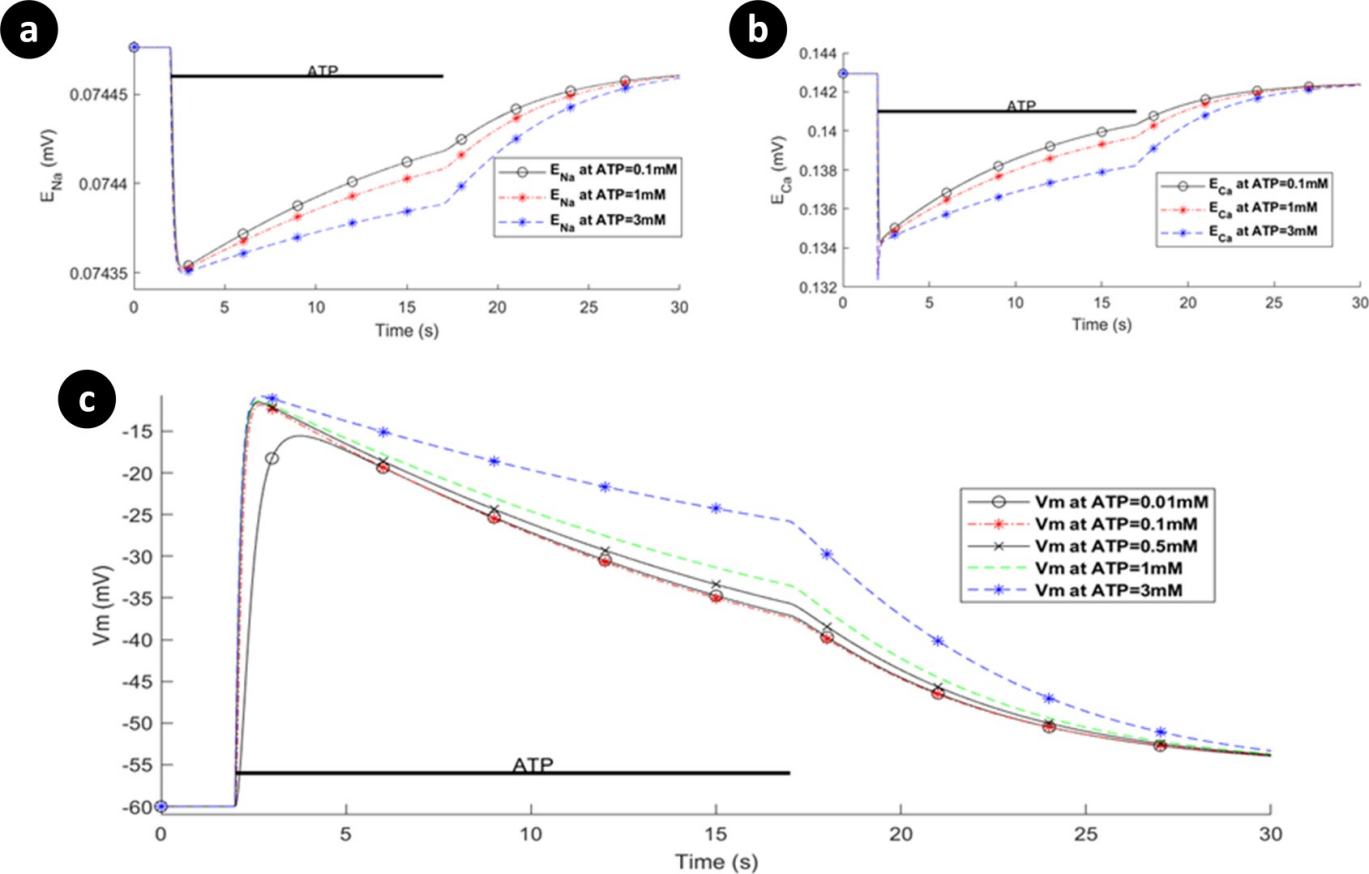
Dynamics of the reversal potentials associated with (a) Ca^2+^, (b) Na^+^, and (c) the membrane potential as a function of ATP levels when the P2X_7_ receptor is turned off.

Note that the mode of operation for the NCX is, to a good approximation, unaffected by influx/efflux of Na^+^ currents since the baseline $\left[{Na}_{i}^{+}\right]$ in the cytoplasm is high (8mM). In contrast, the mode of operation of the NCX is much more sensitive to the $\left[{Ca}_{i}^{2+}\right]$ in the cytoplasm as this quantity changes much more significantly by the ATP stimulus because the baseline $\left[{Ca}_{i}^{2+}\right]$ in the cytoplasm is low (45nM); *V*_*m*_ also drives the NCX. Fig 10 shows the absolute currents carried by the P2X_4_, NCX, PMCA and leak channels for ATP levels of 0.1mM, 1mM and 3mM. Prior to the onset of the ATP stimulus the Na^+^ leak current is negated by NCX extruded Na^+^ efflux and the Ca^2+^ efflux by the PMCA is negated by the NCX operating in reverse mode and calcium leak across the membrane via the Ca^2+^ leak channel. As the ATP stimulus is applied, the P2X_4_ receptor is activated and both Ca^2+^ and Na^+^ current flows into the cytoplasm. In contrast to the P2X_7_ current (see Fig 5), the current through the P2X_4_ (see Fig 7) is more transient in nature, reflecting the behaviour of the biological P2X_4_ receptor. The sharp rise in Na^+^ influx by the P2X_4_ results in an associated rise in the Na^+^ leakage current and *V*_*m*_, with the NCX extruder driven deeper into reverse mode. Also, the sharp rise in Ca^2+^ influx resulting from P2X_4_ activation causes additional efflux of Ca^2+^ via the PMCA but this is somewhat negated by the influx of Ca^2+^ by the NCX, where the latter is driven by hyperpolarisation of *V*_*m*_ and fluctuations in the $\left[{Ca}_{i}^{2+}\right]$ in the cytoplasm. As in the case of the P2X_7_, the reversal potential for Na^+^ (Fig 11) is virtually unaffected due to the high $\left[{Na}_{i}^{+}\right]$ baseline in the cytoplasm but the reversal potential for Ca^2+^ (Fig 11) changes much more significantly because of the low $\left[{Ca}_{i}^{2+}\right]$ baseline in the cytoplasm.

**Fig 12:**
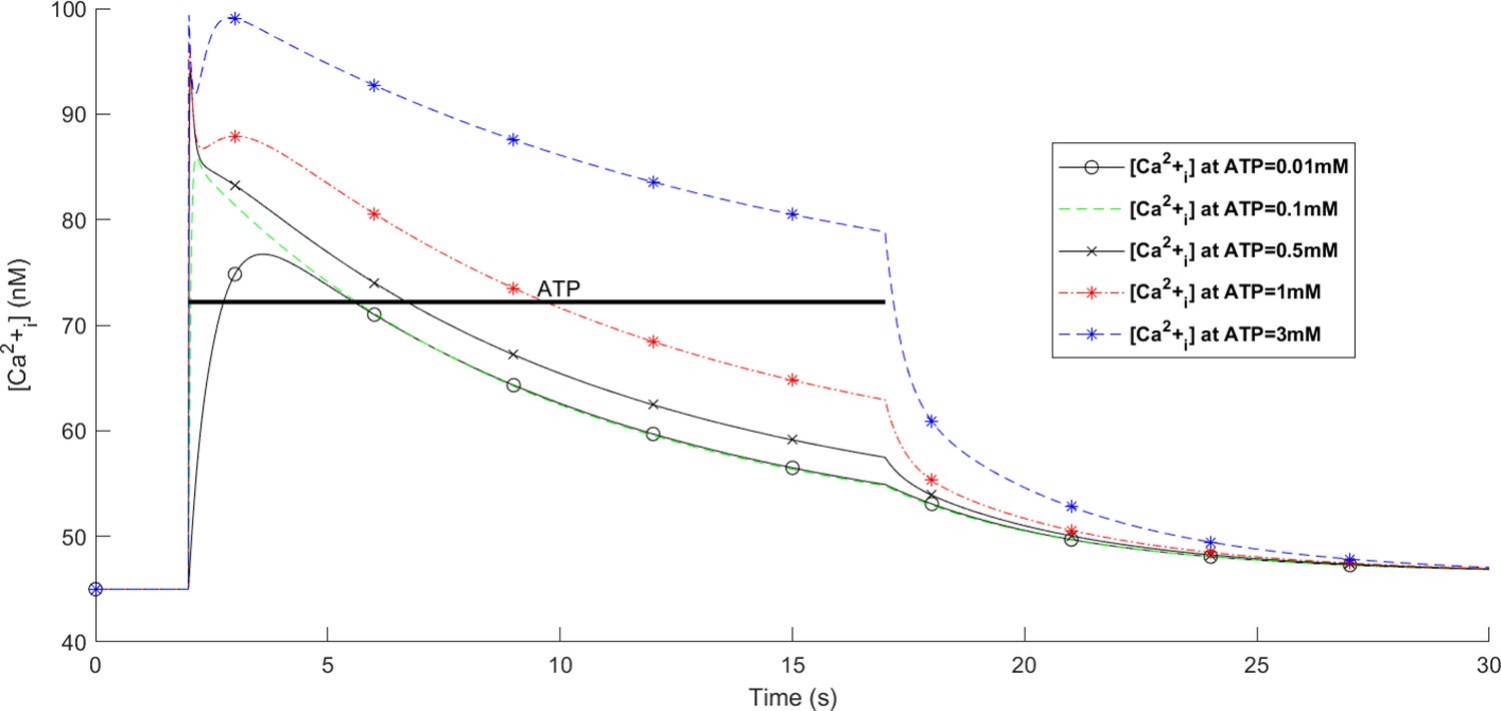
Intracellular Ca^2+^ transients from simultaneous activation of both hP2X_4_ and hP2X_7_ receptors.

A holistic view of $\left[{Ca}_{i}^{2+}\right]$ transients by concurrent stimulation of both hP2X_4_ and hP2X_7_ receptors is given in Fig 12. A significantly distinguished pattern is observed in Fig 12 for ATP = 1mM and ATP = 3mM. As seen in the results of Fig 8, both the Ca^2+^ and Na^+^ currents continue towards saturation while the ATP stimulus is sustained. In contrast, for the P2X_4_ (Fig 10) both the Ca^2+^ and Na^+^ current behave more like a transient (effecting *V*_*m*_) and decay over time irrespective of the level or duration of the ATP stimulus. It is therefore concluded that the kink in the curves at ATP levels of 1mM and 3mM in Fig 12 are because of the fast fall off in $\left[{Ca}_{i}^{2+}\right]$ due to the decrease in the Ca^2+^ current through the P2X_4_ and the relatively slow rise in $\left[{Ca}_{i}^{2+}\right]$ due to the increasing Ca^2+^ current through the P2X_7_. When these two $\left[{Ca}_{i}^{2+}\right]$ cross over (at a comparable concentration) a kink is formed (momentary rise in $\left[{Ca}_{i}^{2+}\right]$ followed by a slow fall off in $\left[{Ca}_{i}^{2+}\right]$). Essentially, both receptors have direct influence on the total P2X-mediated $\left[{Ca}_{i}^{2+}\right]$ dynamics where P2X_4_ receptor dominates the $\left[{Ca}_{i}^{2+}\right]$ at the onset of the ATP stimulus but thereafter the P2X_7_ receptor maintains a high level of $\left[{Ca}_{i}^{2+}\right]$ in the cytoplasm until the removal of the ATP stimulus. A wide spectrum of experimental data confirms this theoretical finding where several P2 (i.e. P2X and P2Y variants) receptors are simultaneously triggered by extracellular nucleotides like ATP. Activation of P2X_4_R and P2X_7_R results in intricate $\left[{Ca}_{i}^{2+}\right]$ kinks for 1mM and 3mM of ATP in rat microglia [38]. Other work on microglia and astrocytes reveal kink-style behaviour of $\left[{Ca}_{i}^{2+}\right]$ [16,45–47] and such dynamics are also observed in other animal and human cell lines including those experimentally reported in [48–53]. Note that no kink appears for ATP < 1mM because the initial rise in current is dominated by current flow through the P2X_7_.

**Fig 13:**
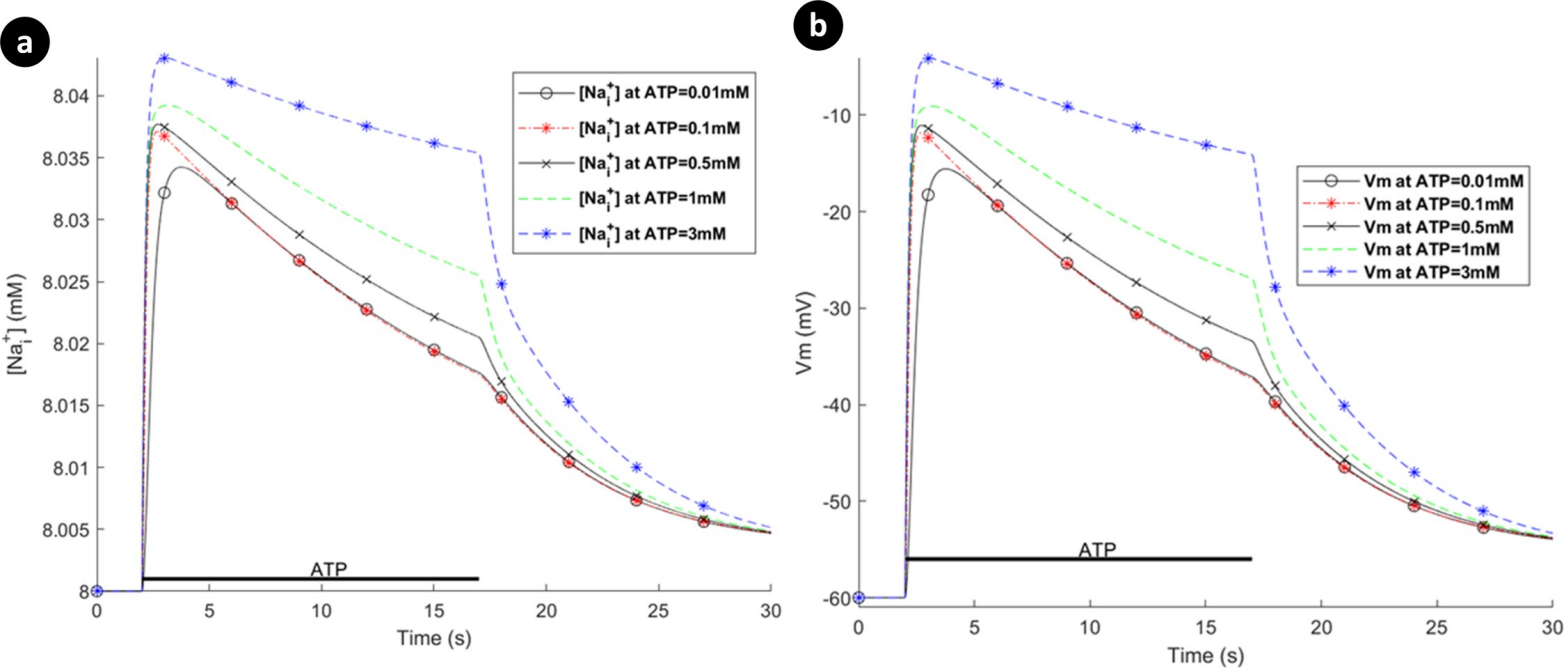
(a) Intracellular $\left[{Na}_{i}^{+}\right]$, and (b) the membrane voltage dynamics resulting from simultaneous stimulation of hP2X_7_ and hP2X_4_ receptors stimulation.

Fig 13 shows the $\left[{Na}_{i}^{+}\right]$ and *V*_*m*_ dynamics. As is evident, both $\left[{Na}_{i}^{+}\right]$ and *V*_*m*_ graphs have similar shapes in contrast to $\left[{Ca}_{i}^{2+}\right]$ counterparts, as their dynamics are dictated by the action of the P2XRs. It is because NCX channels mainly control the flow of Na^+^ cations across the membrane as microglia are reported not to express Na^+^/K^+^-ATPases [7]. The behaviour of *V*_*m*_ indicate that microglia depolarise rapidly, which is also consistent with experimentally reported data for rate microglia [42]. Emerging evidence shows that the dynamic membrane potential, *V*_*m*_, plays key roles in regulation/upregulation of biological processes such as cell migration and proliferation [54]. Therefore, the model may be made more biologically plausible by considering voltage-gated Na^+^ channels and $\left[K_{i}^{+}\right]$ dynamics. It is worth noting that varying the morphological parameters of the model such as *S*_*p*_ and *V*_*p*_ only alters the predicted $\left[{Ca}_{i}^{2+}\right]$ amplitudes slightly and so does not significantly influence the overall response of the Ca^2+^ dynamics (see S3 Text).

**Fig 14:**
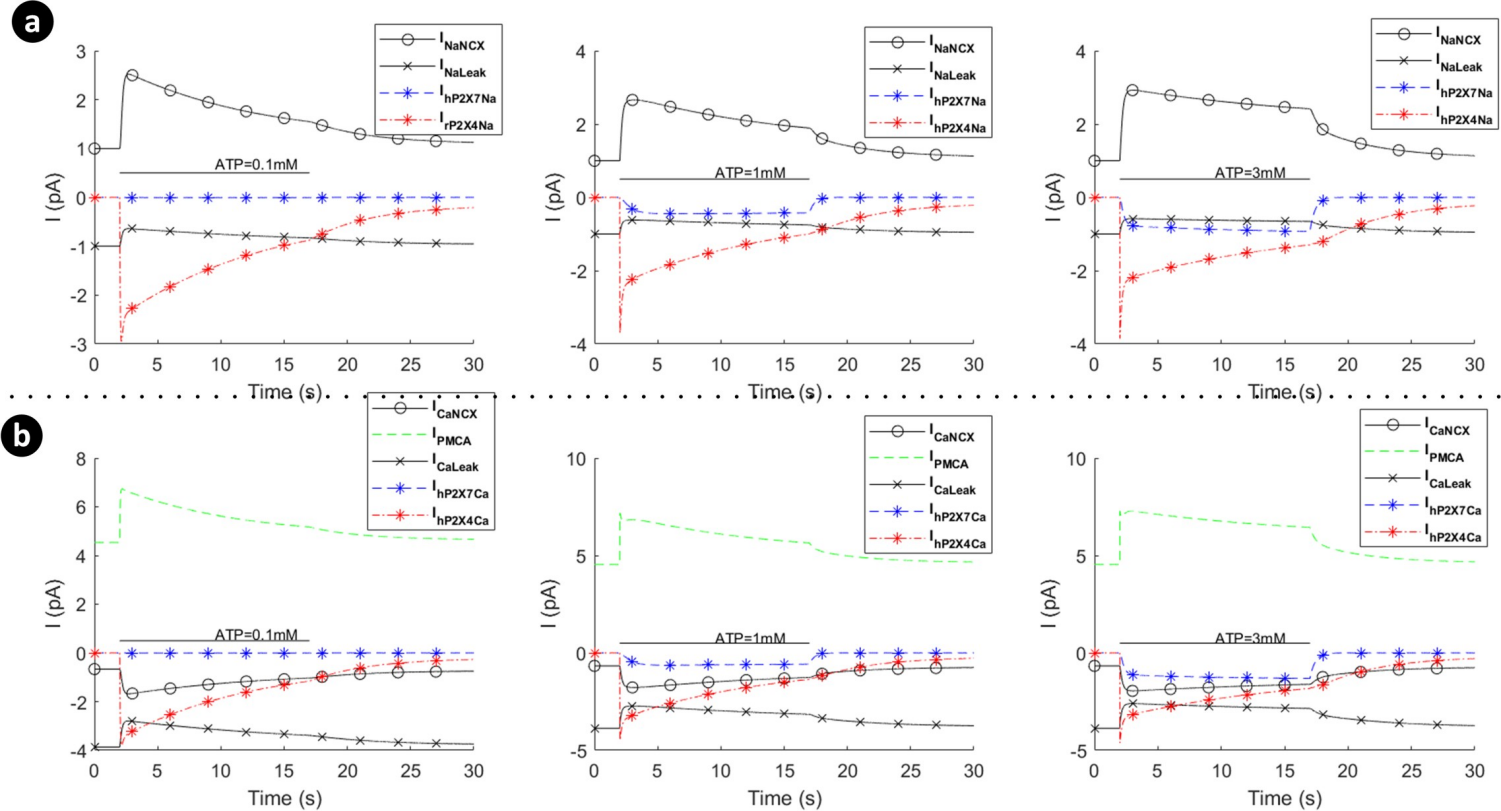
Dynamics of absolute currents via simultaneous activation of hP2X_4_ and hP2X_7_ receptors. Top (a) and bottom (b) panels quantify the Na^+^ and Ca^2+^ components respectively of the model for 0.1mM, 1mM and 3mM ATP.

In contrast to the current profile outlined in Fig 8 for the hP2X_7_ only and in Fig 10 for the hP2X_4_ only, the marked difference when both receptors are activated simultaneously (as seen in Fig 14), is the domination of the hP2X_4_ receptor current at high levels of ATP, while the hP2X_7_ stays constant at higher ATP levels and over a longer duration. In addition the kink in the concentration profile for Ca^2^ directly correlates with the initial fall off in the Ca^2+^ current, carried by the PMCA, due to the decaying Ca^2+^ influx by hP2X_4_ and the increasing Ca^2+^ influx by hP2X_7_ (see curves for 1mM and 3mM ATP levels).

To summarise, it is inferred that the very transient behaviour of the kinks in Fig 12 is indicative of cooperative dynamics between activation of several P2XRs, PMCA and NCX, where NCX mainly derives the $\left[{Na}_{i}^{+}\right]$ extrusion. As such, *V*_*m*_ is similar to Na^+^ dynamic in Fig 13 because of a high baseline of $\left[{Na}_{i}^{+}\right]$, and leak channels keep microglia sensitive enough to changes in their microenvironment.

### Sensitivity analysis of the mathematical model

As demonstrated throughout this paper P2X-mediated Ca^2+^ dynamics are primarily controlled by ATP-gated P2XRs, NCX, PMCA and leak channels. This section carries out sensitivity analysis (SA) of the model parameters to elucidate the robustness of the model responses [55]. It is also a powerful means whereby the parameters or components that substantially contribute to the model predictions can be determined. The sensitivity analysis results can provide a detailed insight into the model parameters for future model refinements when new experimental data becomes available in order to calibrate the model at different levels of agonist. Sensitivity can also be viewed as the inverse of robustness where sensitivity of parameters give rise to a quantitative estimate in deviations of model outcomes arising from perturbation of model parameters. Since the behaviour of high-dimensional biochemical reaction networks is often dominated by a small number of parameters, SA helps identify these parameters that can be investigated in further studies.

In this section, two different methods of SA ar are employed to obtain additional information regarding the P2X model parameters: these methods are local and global sensitivity analysis (LSA and GSA). In LSA, a single parameter (where others are kept constant) is varied and the effect of this perturbation is considered. This process is repeated for all parameters. In contrast to LSA, GSA techniques employ simultaneous pertubations to model parameters, and changes in the model output are monitored. GSA is capable of detecting relationships between a set of parameters. Logarithmic sensitivity analysis (LSA) [56], was initially used to investigate the influence of the model parameters on the model output. LSA is dimensionless and therefore allows comparison of the results conveniently. If *y* is a single response of a system of ODEs, LSA of this variable is defined as

$$S_{y}=\frac{\partial ln\;y(t,p_{i})}{\partial ln\;p_{i}}=\frac{p_{i}}{y(t,p_{i})}\times \frac{\partial y(t,p_{i})}{\partial p_{i}}$$ (18)

where *p*_*i*_ denotes the *i*’th parameter. Partial derivative of *y* in Eq (18) with respect to *p*_*i*_ can be approximated by using forward finite difference (FD) [55] as follows

$$\frac{\partial y(t,p_{i})}{\partial p_{i}}\approx \frac{y(t,p_{i}+\Delta p_{i})-y(t,p_{i})}{\Delta p_{i}}$$ (19)

where *Δp*_*i*_ is a notation for a small change of the parameter *p*_*i*_.

**Fig 15:**
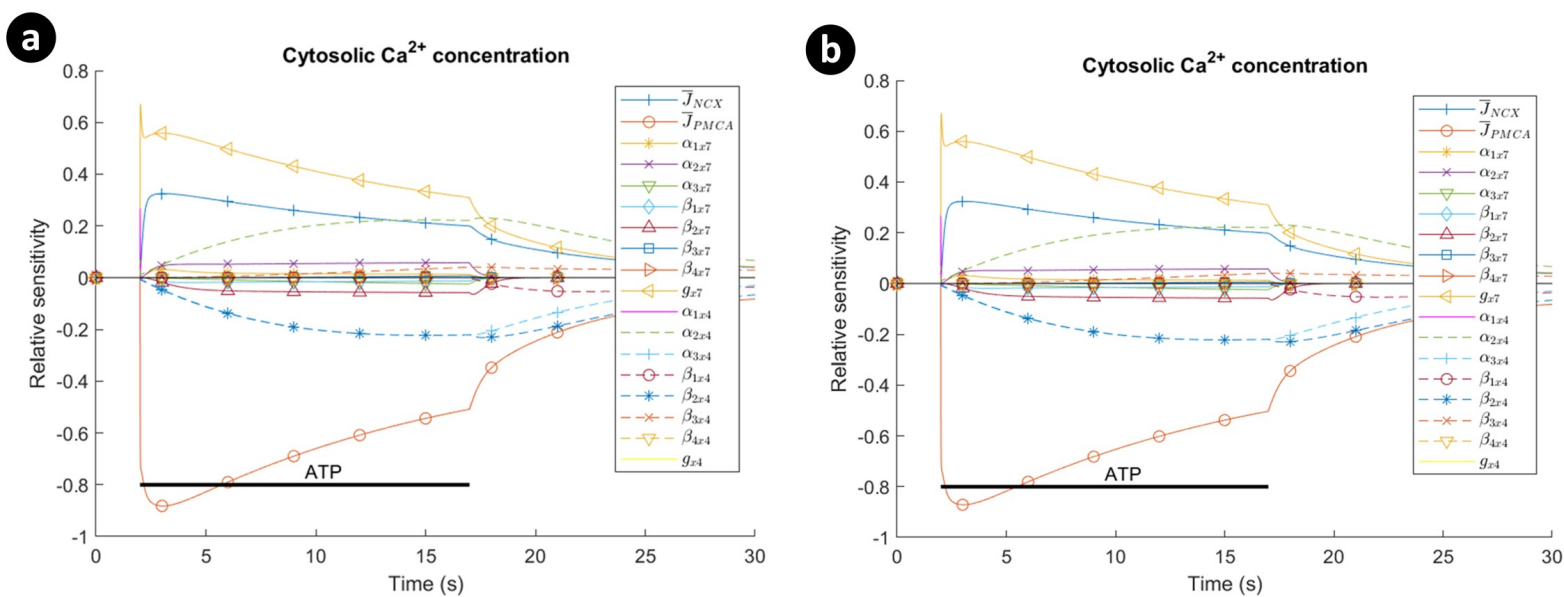
The sensitivity analysis of the intracellular Ca^2+^ for ATP = 1mM with respect to maximum NCX current density (${\bar{J}}_{NCX}$), maximum PMCA current density (${\bar{J}}_{PMCA}$), and the parameter sets of hP2X_7_ and hP2X_4_ receptors: (a) 0.1% perturbation of the model parameters was employed, namely, *Δp*_*i*_ = 0.001×*p*_*i*_, and (b) 1% perturbation of the model parameters was used in this case, viz., *Δp*_*i*_ = 0.01×*p*_*i*_. Horizontal bar show the duration of agonist exposure.

As a general rule of understanding sensitivity analysis robustness, if the results do not change significantly after the fitted model parameters are perturbed then the sensitivity analysis is said to be robust [55]. For this study, the sensitivity analysis was carried out on the cytosolic Ca^2+^ concentration with respect to the maximum NCX current density (${\bar{J}}_{NCX}$), the maximum PMCA current density (${\bar{J}}_{PMCA}$), and the parameter sets of the P2X models by stimulating the model under 1mM ATP. Fig 15 illustrates a plot of Eq (18) for parameter pertubations of 0.1% and 1% (note that *x7* and *x4* subscripts represents P2X_7_ and P2X_4_ in Fig 15, respectively). Both graphs are similar and this verifies the sensitivity analysis robustness of the fitted model. More importantly, the uptake of Ca^2+^ is mainly affected by ${\bar{J}}_{NCX},\;{\bar{J}}_{PMCA}$, *g*_*x7*_, *α*_*2x4*_ and *β*_*2x4*_. The intracellular Ca^2+^ concentration exhibits negative and positive sensitivity to PMCA and NCX parameters respectively. S4 Text considers the impact of different parameter settings on the model predictions. S6 Text illustrates the sensitivity graphs in Fig 15 individually.

**Fig 16:**
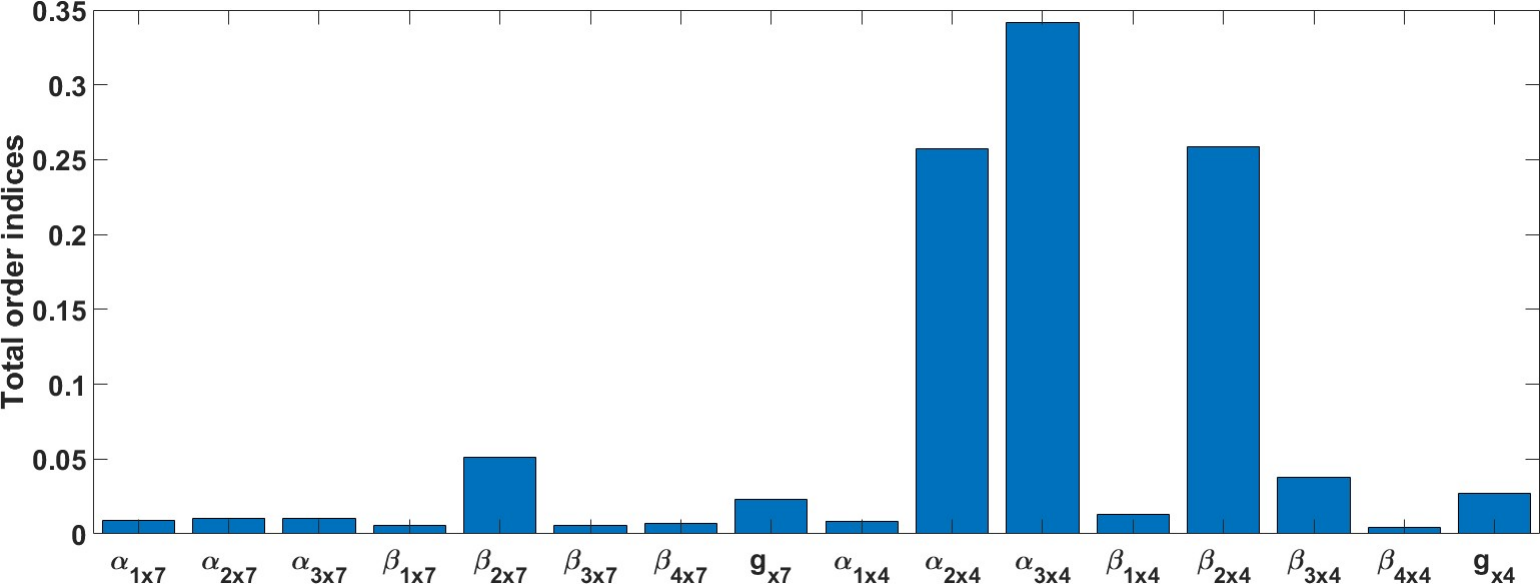
Total order Sobol indices of the intracellular Ca^2+^ model at ATP = 1mM for the peak duration.

Several techniques exist to carry out GSA. Sobol’s method is used in this section, which is a variance-based method [57]. The method does not make any assumption for input and output relationships of a model. The variance of the model output is decomposed into a combination of variances. A Joint Probability Function (PDF) is employed to make the Sobol’s terms. A MATLAB implementation of this method developed by [58] was used to perform GSA and study the effect of simultaneous changes of model parameters on cytosolic Ca^2+^ for ATP = 1mM. Fig 16 shows the computed Sobol’s total indices for the peak duration. A peak duration is defined as the time interval at which the transient values of the model output is greater than or equal to the mean of the output throughout the simulation time. According to Fig 16 for total Sobol indices, the five most dominant parameters are *α*_*3x4*_, *α*_*2x4*,_ *β*_*2x4*_, *β*_*2x7*_ and *β*_*3x4*_ in decreasing order. *β*_*2x7*_ and *g*_*x7*_ have more influence on the output among all other parameters of the P2X_7_ model. The effect of both P2X conducntances is nearly close, while there is a significant variation for most of the corresponding parameters of both P2X models. *β*_*2x7*_ and *β*_*2x4*_ have the biggest impact on the output among all other backward rate coefficients for each individual model. Interestingly, the global sensitivity of *g*_*x7*_ in Fig 16 is significantly less influential than its local sensitivity illustrated in Fig 15. These LSA and GSA results show that the parameters of the P2X_4_R model are mostly dominant in the model sensitivity as a whole.

## Discussion

Neurotransmitters can modulate intracellular Ca^2+^ and Na^+^ via activation of microglial receptors, which regulate their homeostatic mechanisms [7]. Microglia express P2X_4_ and P2X_7_ purinergic receptors which impact local Ca^2+^ homeostasis. Mathematical models that result in a formal description of agonist binding to purinergic receptors with receptor activation, provide unique tools to unravel the underlying regulatory dynamics of intracellular ionic concentrations. In this research a minimal computational model for Ca^2+^, Na^+^ and *V*_*m*_ dynamics of microglia was developed. The binding of ATP to P2XR on microglial processes leads to receptor activation, with the associated influx of Ca^2+^ and Na^+^. This results in a rise mainly in $\left[{Ca}_{i}^{2+}\right]$ and *V*_*m*_ hyperpolarising, leading to activation of the PMCA, NCX and leak channels: it is worthwhile noting that this model allows a detailed investigation into the interplay between the P2XRs, pumps, extruders, membrane voltage and leak channels. The model predictions show that activation and deactivation of P2X_4_ and P2X_7_ receptors in microglia generate monophasic and biphasic shapes in conformance to microglia-specific P2X data or other cell-specific data at lower and higher ATP concentrations. Particularly, for the P2X_7_ receptor, there are two different phases of current initialisation and current facilitation. Simulation observations show that monophasic and biphasic transients are also present in single cell Ca^2+^ and Na^+^ profiles. The model indicates that P2X_7_ receptor maintains significant currents and therefore effect ionic concentrations much more than P2X_4_ at higher levels of ATP. These data have implications for brain pathology, where elevated levels of ATP are evident [59,60]. The relative contributions of the P2XRs studied here may provide insight into therapeutic strategies targeting either receptor. For example, P2X_7_ antagonism has been suggested as a therapeutic intervention for a range of CNS disorders [61–63]; here the modelling data present a read-out of this pharmacological intervention in relation to ionic homeostasis and microglia function. In addition, there are implications from the model with respect to microglial function where their levels of the P2XRs are known to be altered (e.g. Parkinson’s disease; [64]).

The model demonstrates that Ca^2+^ dynamics not only rely on different gating property of P2XRs as a function of ATP pulse protocol but also on the opposing Ca^2+^ currents carried by PMCA and NCX: the latter being continually in the forward mode due to the hyperpolarisation of *V*_*m*_ and $\left[{Ca}_{i}^{2+}\right]$ fluctuations in the cytoplasm. At ATP levels of 1mM and 3mM, unexpected kinks are observed in the concentration of Ca^2+^ regulated by $\left[{Na}_{i}^{+}\right]$ and *V*_*m*_. This kink is a direct consequence of the fast fall off in $\left[{Ca}_{i}^{2+}\right]$ due to the continual decrease in the Ca^2+^ influx, via the P2X_4_ competing with the slow rise in $\left[{Ca}_{i}^{2+}\right]$ due to the increasing Ca^2+^ influx via the P2X_7_. Hence there exists a cross over in the $\left[{Ca}_{i}^{2+}\right]$ and a kink appears followed by a gradual decrease in $\left[{Ca}_{i}^{2+}\right]$: the P2X_4_ receptor dominates the $\left[{Ca}_{i}^{2+}\right]$ at the onset of the ATP stimulus and thereafter the P2X_7_ receptor maintains a high level of $\left[{Ca}_{i}^{2+}\right]$ in the cytoplasm until the removal of the stimulus. The abstraction level of the proposed model facilitates the reproduction of meaningful responses arising from the interplay between the P2XRs, PMCA, NCX and leak channels.

The current experimental data shows a total cellular current where the contribution to the total current comes from Ca^2+^ and Na^+^ influx, and K^+^ efflux. With regards to the latter, the mechanisms underpinning K^+^ efflux are poorly understood and are therefore not considered in this paper. However, the influx of Na^+^ will affect the NCX and therefore have a downstream effect on Ca^2+^. Because of this, the contribution to the Na^+^ concentration in the cytoplasm by the P2XRs was considered for a better understanding of the observed Na^+^ and Ca^2+^ dynamics. To the best of our knowledge, there is no theoretical or experimental study of activation of multiple ionotropic receptors in human microglia. Cultured human microglia experiments failed to isolate P2X_4_ currents [22] nor did they provide data on the interaction between different P2XRs. However, the model presented in this paper does provide insight into both of these receptors and how they collectively contribute in terms of Ca^2+^ and Na^+^ dynamics. Moreover, the simulation results in this paper used human data in so far as possible and can inform experimentalists as to the role of Na^+^ influx and how this can affect downstream Ca^2+^ dynamics.

The proposed computational framework provides a strong foundation that can be extended towards a more complete understanding of microglial function, particularly, *in situ* and *in vivo*. The model can also be refined as new experimental data becomes available and to this end the expansion in human microglia research through the use of induced pluripotent stem cells is of interest [65]. Additionally, the function of the P2Y_12_ receptor will be the focus of future work, as these are highly expressed in activated microglia [66]. It has been shown that P2Y_12_ actively participate in triggering the PI3K pathway through Ca^2+^ uptake from the extracellular space [8,67]. Other future work on extending the model proposed here is the inclusion of distinct types of K^+^, and voltage-gated Na^+^ channels, which have rapid kinetics and depend on *V*_*m*_. The biophysical model developed in this paper will improve our quantitative understanding of P2X-mediated microglial physiology.

## Supporting information

S1 Text Non-linear curve fitting based on evolution strategy. It provides details on curve fitting of the P2X model.
(DOCX)

S2 Text Model comparisons. It shows two existing P2X models cannot capture microglial P2X data well.
(DOCX)

S3 Text Effect of morphological changes on cytosolic calcium dynamics. By varying the underlying morphological parameters of the model, it is concluded that the model can reliably predict the general behaviour of P2X-medited calcium signalling virtually independent of the surface area and volume of microglia.
(DOCX)

S4 Text Effect of different model parameter settings on predictions. It mainly discusses the model fitting robustness.
(DOCX)

S5 Text Contribution of model components and parameters to represent P2XR biological data. It discusses the necessity of model elements in a quantitative manner.
(DOCX)

S6 Text Separate graphs for local sensitivity analysis of the model. It provides a better illustration of multiple sensitives given in Fig 15.
(DOCX)

S1 Data It includes raw data extracted from graphical experimental data sets presented in [22,23] for both P2X receptors and used in the fitting process. (ZIP)
