# Supplementary material for: Mathematical modelling of human P2X-mediated plasma membrane electrophysiology and calcium dynamics in microglia
Source: PLoS Comput Biol. 2021 Nov 1;17(11):e1009520. doi: 10.1371/journal.pcbi.1009520 (PMC8584768; doi:10.1371/journal.pcbi.1009520)
Supplement: S1 Text — It provides details on curve fitting of the P2X model. (DOCX) [file pcbi.1009520.s001.docx]

**S1 Text. Non-linear Curve Fitting based on Evolution Strategy**

Parameter estimation is a kind of inverse problems, where the parameters cannot be directly determined are approximated to comply with the best fit of the biophysical model to experimental data. Parameters of the P2X model, i.e. kinetic rate constants, along with initial conditions of its system of ODEs describe dynamics of the biophysical model. A significant task in this modelling approach is to estimate the model parameters through experimental data, many of which are usually unknown. Complex models have many state variables that are either difficult to measure experimentally or have dependency on other undiscovered pathways (the non-identifiability issue). In a complex biological system or the P2X models in this paper, a few of observables can be determined empirically (e.g., the whole-cell patch-clamp P2X currents are only available), therefore, traditional curve fitting methods such as non-least squares fail. Alternative optimisation algorithms have been proposed to address the above problems.

Evolution strategies (ES) are among those algorithms that are stochastic and derivation-free. ES is often used for numerically optimising a non-linear/non-convex minimisation problem. Evolutionary methods are inspired by natural selection that imitates biological evolution. Population of separate solutions are iteratively updated. Individuals are selected from current population and nominated as parents for the next iteration for generating new children. The algorithm is repeated until an optimal solution is reached for the population. Multivariable normal distribution is used to sample new optimal solutions. Covariance Matrix Adaptation Evolution Strategy (CMA-ES) is a type of ES ] that updates the covariance matrix of the distribution. Iteration over the covariance matrix results in a second-order learning of the underlying cost function. CMA-ES can be seamlessly used to find appropriate values of the model parameters in terms of rate constants.

Parameter estimation of the model was carried out by CMA-ES which is also available as a MATLB library ]. Algorithm 1 portrays three essential phases in curve fitting of our model. Data points are prepared for fitting to the model at the first phase. Experimental data is fed into a spline interpolation algorithm, so a uniform distribution of data points can be obtained. At the second stage, environmental setup of the underlying GA (genetic algorithm) optimiser is carried out, for example, choosing an appropriate estimate of the population size, lower and upper bounds at which the parameters are estimated (note that all parameters correspond to positive values because they are mostly indicating the kinetic rates of the P2X model), the radius of search (the value of 0.5 was used here) and a function callback.

The estimation process interacts with the CMAES library through a callback function in where the curve fitting handler is invoked on every GA iteration. Finally, the third phase performs the actual fitting. In each iteration, the ODE equations—Eqs. [1], [2], [3] and [4]—must be numerically solved. To improve the stability of numerical integration, we utilise Jacobian matrix due to non-linearity of model equations. The distance between experimental data and the fitted model response must be calculated in each iteration. A variety of cost functions for this purpose can be defined whose performance may depend on specific problems. Mean Squared Error (MSE) was chosen as a basic loss function that can result in stable and good fits to both hP2X_7_R and rP2X_4_R data. The fitting process terminates when a specific tolerance denoted as *tol* in Algorithm 1 is met (we used$tol={10}^{-6}$). After completion of the optimisation process, final MSE values became 0.105 and 0.0301 respectively hP2X_7_R and hP2X_4_R for 500 fitting points.

**
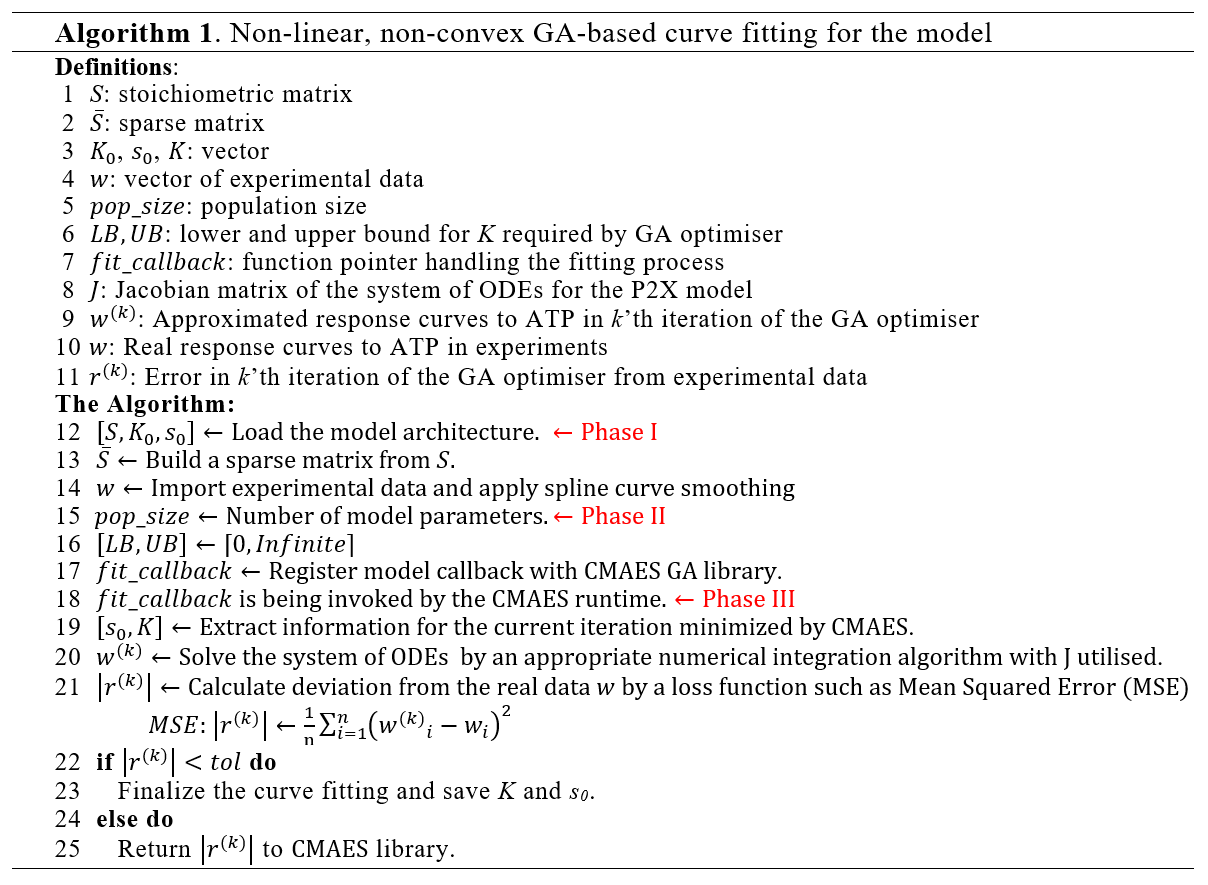
**

**Supplementary References**

1.Hansen N. The CMA evolution strategy: a comparing review. Towards a new evolutionary computation: Springer; 2006. p. 75-102.

2.The CMA Evolution Strategy 2021 [Available from: <http://cma.gforge.inria.fr/index.html>.
