# Supplementary material for: Mathematical modelling of human P2X-mediated plasma membrane electrophysiology and calcium dynamics in microglia
Source: PLoS Comput Biol. 2021 Nov 1;17(11):e1009520. doi: 10.1371/journal.pcbi.1009520 (PMC8584768; doi:10.1371/journal.pcbi.1009520)
Supplement: S2 Text — It shows two existing P2X models cannot capture microglial P2X data well. (DOCX) [file pcbi.1009520.s002.docx]

**S2 Text. Model Comparisons**

We tried several existing models to fit and find reasonable predictions for human microglia as mentioned in the main article. There were two critical issues with existing models. First, they were not capable of capturing a single data set very well. Second, their predictions were not reliable. Two existing models are implemented [1, 2] and their fittings are shown below:.

**First**, we implemented the model from (1) by fitting it to human data for the P2X_7_ receptor (hP2X_7_). The poor fitting is shown in S2 Fig 1.

**(a)**

| 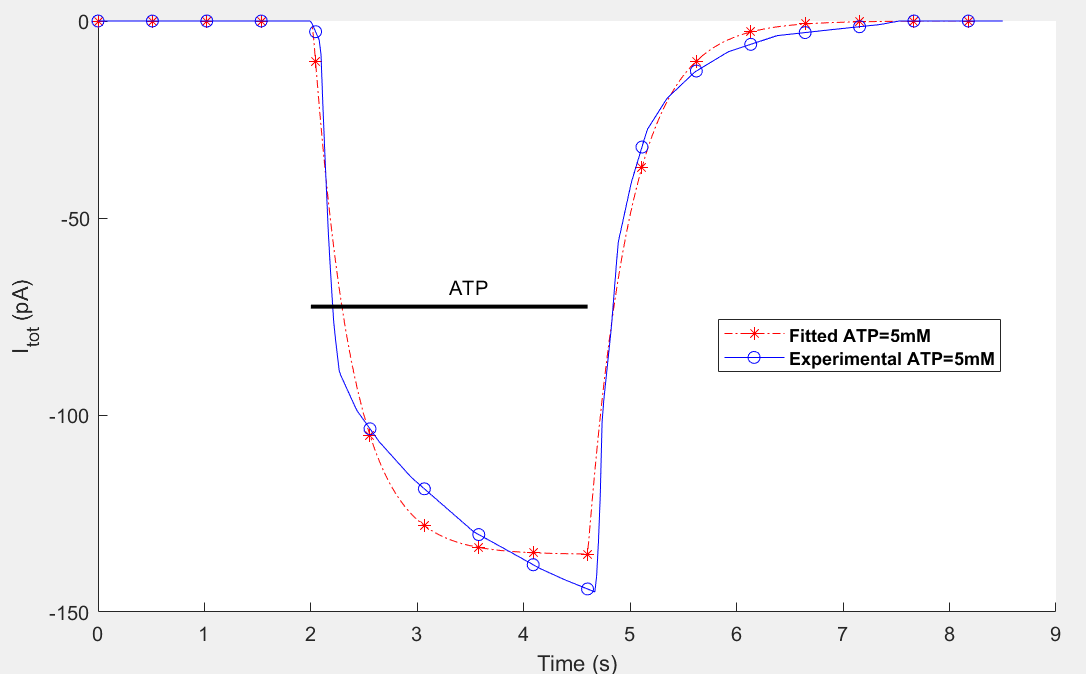 | 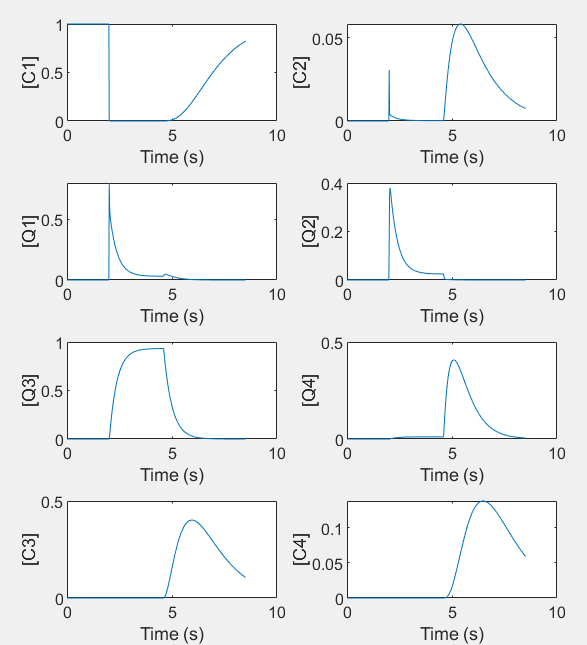  **(b)** |
| --- | --- |

**S2 Fig 1:** (a) Fitting the model in (1) to microglial hP2X_7_ data, and (b) state space of the fitted model.

Furthermore, the predictions of [1] are illustrated in S2 Fig 2.

| 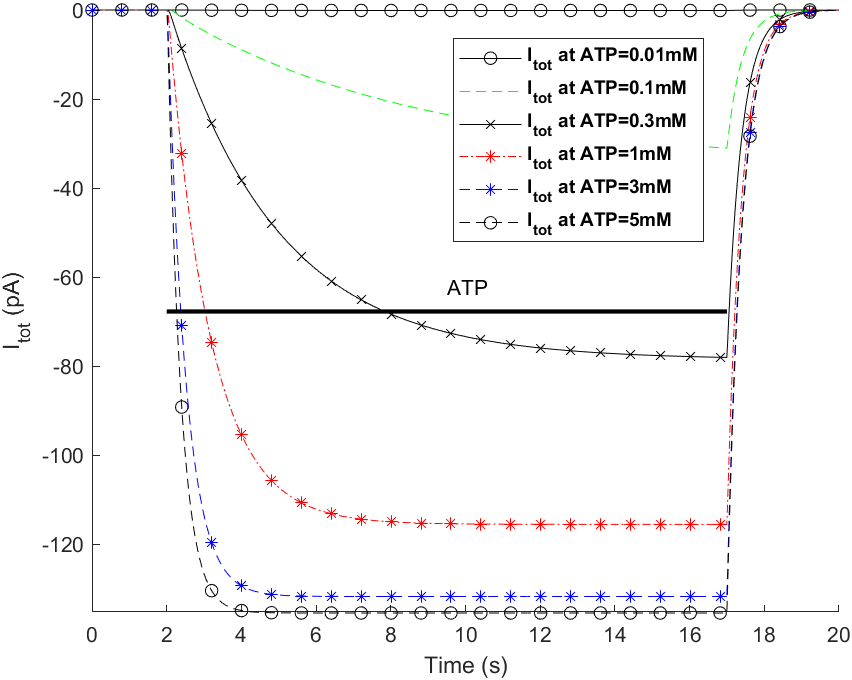 | 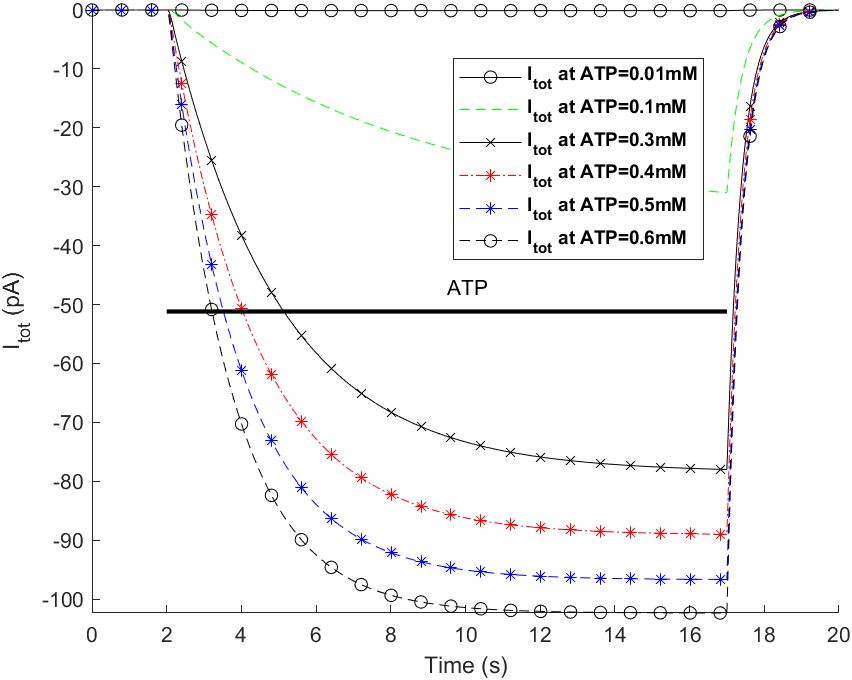 |
| --- | --- |

**S2 Fig 2:** Simulation of the fitted model in S2 Fig 1.

As it can be seen again, the predictions of the model are not reliable for human microglia because at least we expect to see a local minimum at ATP=1mM (see the results section of the main paper in Fig 5(b) and the dissuasion for Fig 7(a)). All the results are not consistent with our results discussed in the paper.

**Second**, as another example, we implemented a model for the rat P2X_4_ receptor from (2). Model simulations for different levels of ATP appear in S2 Fig 3.

| 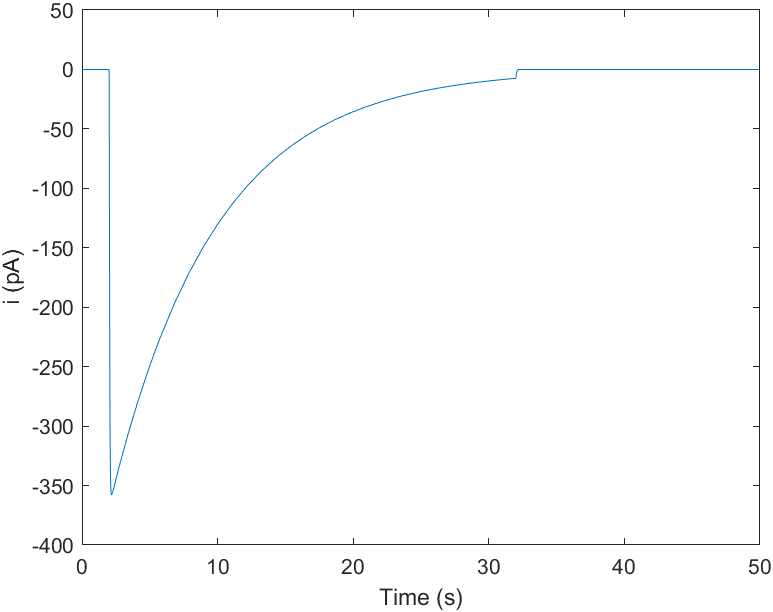  **ATP=0.1mM** | 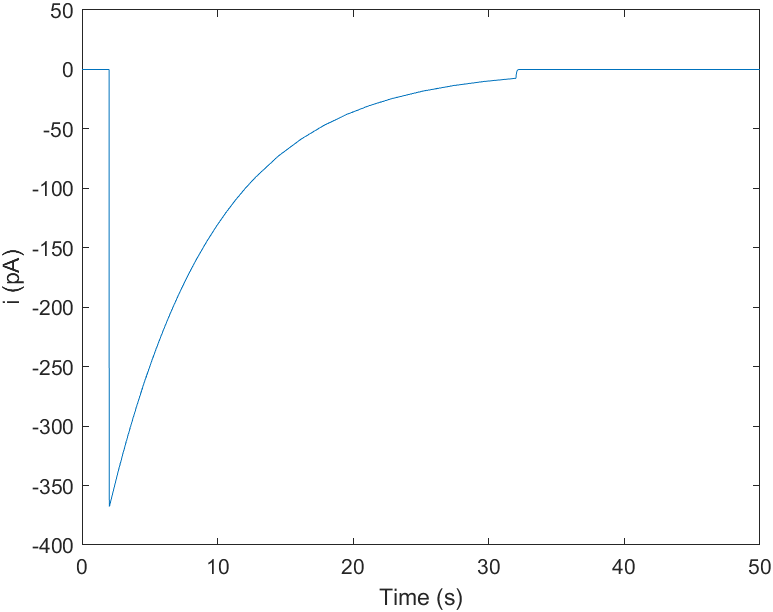  **ATP=1mM** | 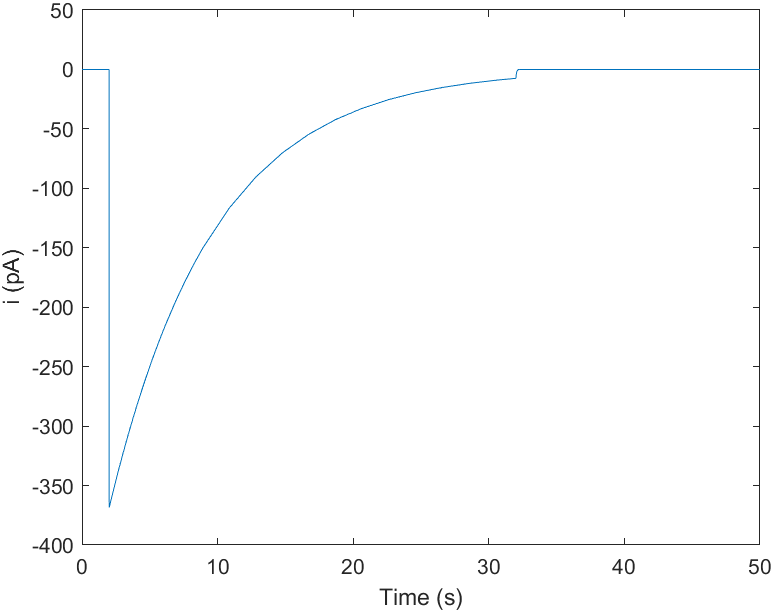  **ATP=3mM** |
| --- | --- | --- |

**S2 Fig 3:** Simulation of the P2X_4_ model in (2) for different levels of ATP.

It is clear that the model predictions in S2 Fig 3 is insensitive to the ATP level so it cannot be used as a good candidate for our microglial human prediction. As we have detailed in the main article, the desentisation of P2X_4_ receptor depends on the amplitude of the stimulus, particularly, in human cell lines.

**Supplementary References**

1. Yan Z, Khadra A, Li S, Tomić M, Sherman A, Stojilkovic SS. Experimental characterization and mathematical modeling of P2X7 receptor channel gating. J Neurosci. 2010;30(42):14213-24.

2. Chun BJ, Stewart BD, Vaughan DD, Bachstetter AD, Kekenes‐Huskey PM. Simulation of P2X‐mediated calcium signalling in microglia. J Physiol. 2019;597(3):799-818.
