# Supplementary material for: Mathematical modelling of human P2X-mediated plasma membrane electrophysiology and calcium dynamics in microglia
Source: PLoS Comput Biol. 2021 Nov 1;17(11):e1009520. doi: 10.1371/journal.pcbi.1009520 (PMC8584768; doi:10.1371/journal.pcbi.1009520)
Supplement: S3 Text — By varying the underlying morphological parameters of the model, it is concluded that the model can reliably predict the general behaviour of P2X-medited calcium signalling virtually independent of the surface area and volume of microglia. (DOCX) [file pcbi.1009520.s003.docx]

**S3 Text. Effect of Morphological Changes on Cytosolic Calcium Dynamics**

Here, the influence of morphological changes on intracellular Ca^2+^ dynamics is investigated. **Firstly**, The surface area and volume of the microglial model was refined to have a spherical shape by assuming a radius of 0.5278μm (1). S3 Fig 1 shows the results when a cube is chosen as compared to a sphere. As seen, morphological alternations slightly change the magnitudes of cytosolic Ca^2+^ concentration. In this case study, the difference between the peak Ca^2+^ amplitude of a cubic and spherical shape is roughly 0.143nM. **Secondly**, we created two different perturbations into the model morphological parameters (1% and 10%) to see how they affect the cytosolic Ca^2+^ responses. The results come in S3 Fig 2 and S3 Fig 3 for changes in $S_{q}$ and $V_{q}$(where responses of the original and perturbed shapes completely overlap). In these two case studies, the difference between the peak Ca^2+^ amplitude of the original shape (cubic) and and its perturbed shape was roughly 1.4210854715202×10^-14^nM and 0nM respectively for S3 Fig 2 and S3 Fig 3. These results likewise imply that the model is robust and its overall behaviour is virtually independent of the underlying morphological phenotype.


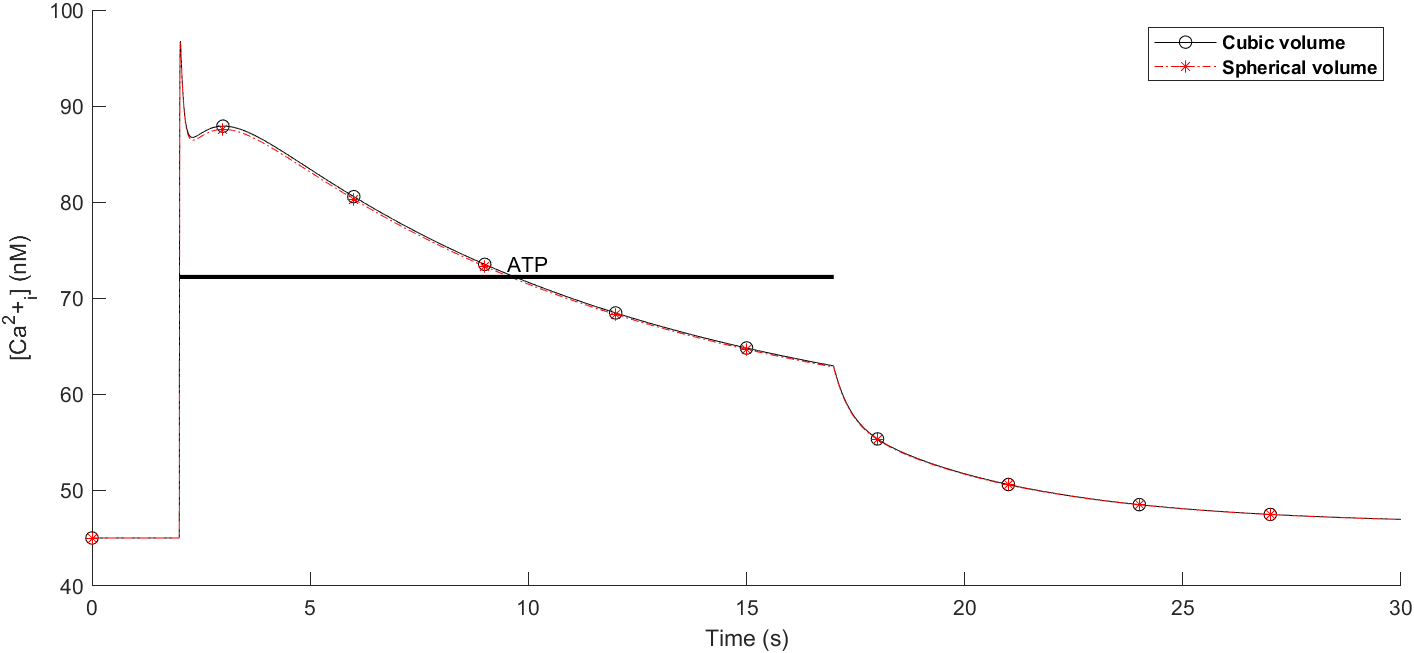


**S3 Fig 1:** Effect of cubic and spherical morphology on intracellular Ca^2+^ transients from simultaneous activation of both rP2X_4_ and hP2X_7_ receptors when ATP is set to 1mM.


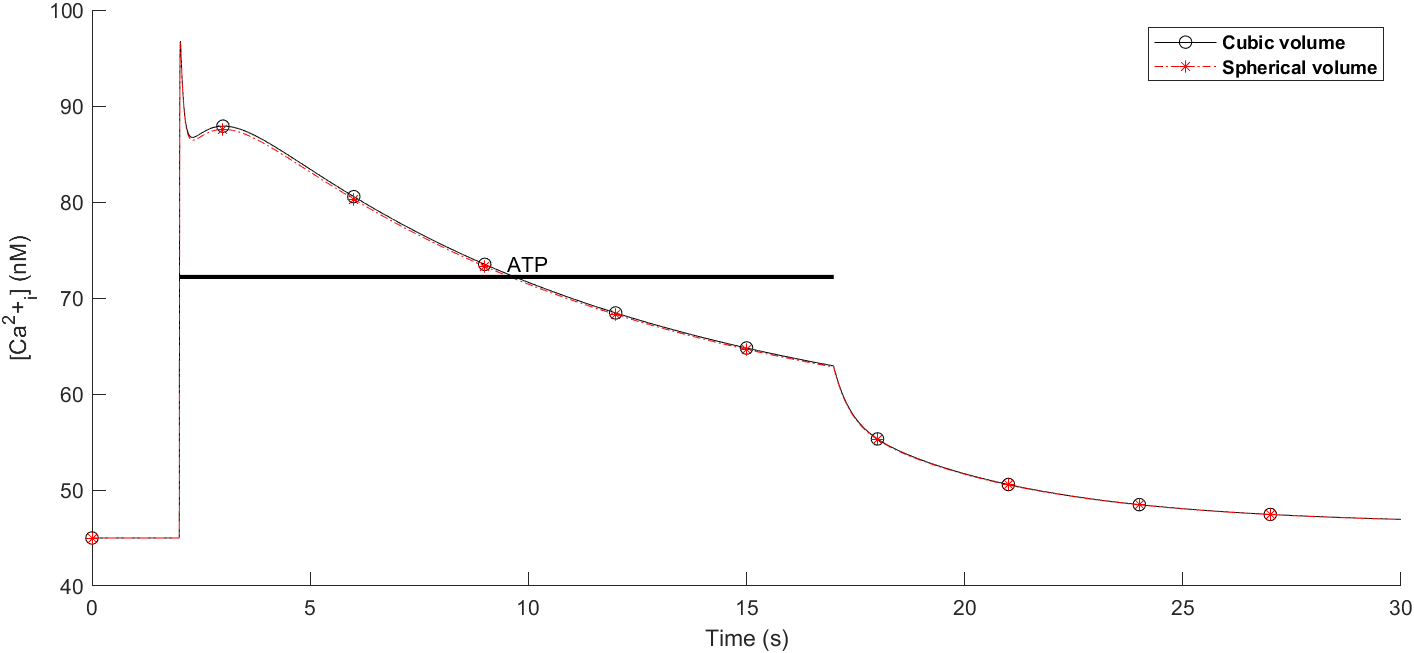


**S3 Fig 2:** Effect of perturbed morphology on intracellular Ca^2+^ transients from simultaneous activation of both rP2X_4_ and hP2X_7_ receptors when ATP is set to 1mM. Note that 1% perturbation of the model morphology was employed, namely, $\Delta S_{q}=0.01\times S_{q}$ and $\Delta V_{q}=0.01\times V_{q}$.


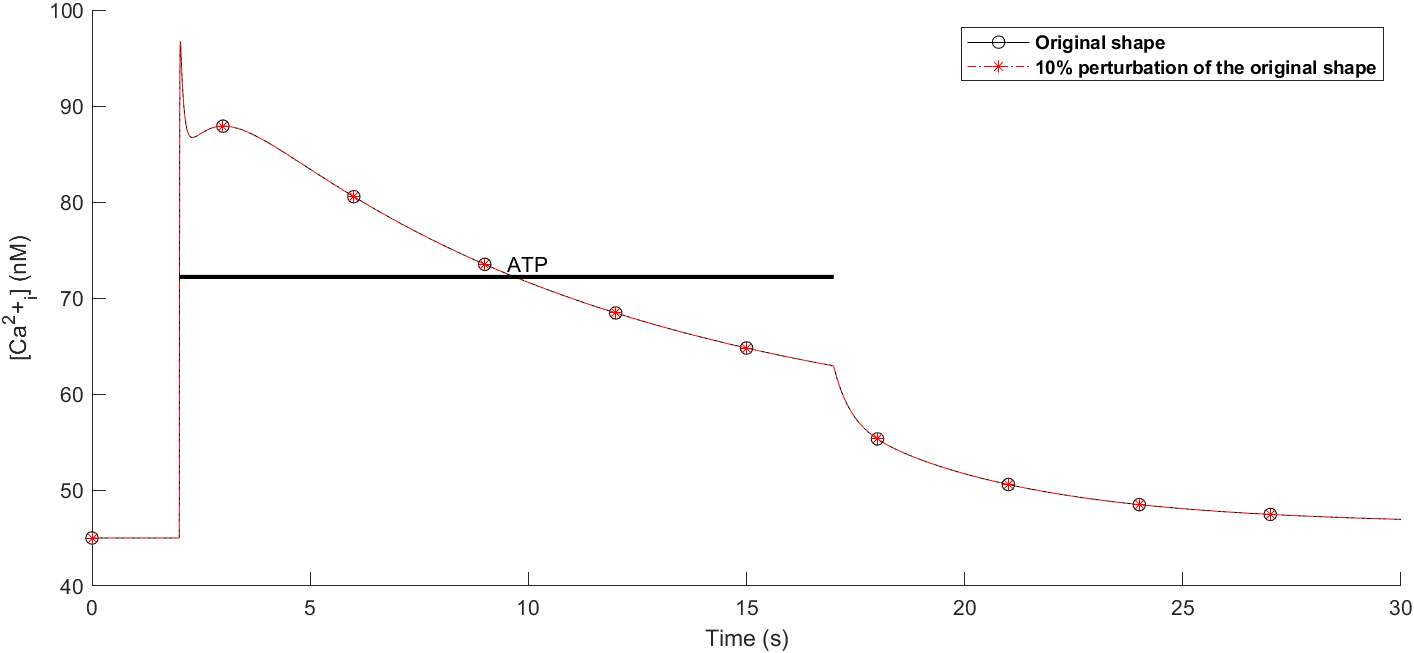


**S3 Fig 3:** Effect of perturbed morphology on intracellular Ca^2+^ transients from simultaneous activation of both rP2X_4_ and hP2X_7_ receptors when ATP is set to 1mM. Note that 10% perturbation of the model morphology was employed, namely, $\Delta S_{q}=0.1\times S_{q}$ and $\Delta V_{q}=0.1\times V_{q}$.

**Supplementary References**

1. Chun BJ, Stewart BD, Vaughan DD, Bachstetter AD, Kekenes‐Huskey PM. Simulation of P2X‐mediated calcium signalling in microglia. J Physiol. 2019;597(3):799-818.
