# Supplementary material for: Mathematical modelling of human P2X-mediated plasma membrane electrophysiology and calcium dynamics in microglia
Source: PLoS Comput Biol. 2021 Nov 1;17(11):e1009520. doi: 10.1371/journal.pcbi.1009520 (PMC8584768; doi:10.1371/journal.pcbi.1009520)
Supplement: S4 Text — It mainly discusses the model fitting robustness. (DOCX) [file pcbi.1009520.s004.docx]

**S4 Text. Effect of Different Model Parameter Settings on Predictions**

Given the nature of GA parameter fitting, several runs of the fitting may produce similar results but very different parameter settings. To investigate this, we ran the fitting several times with different initial seeds and compared the final parameters against each other. We also ran the prediction simulations, which are outside the experimental regime, to compare their predictions. S4 Table 1 shows the fitted parameters for the P2X model for four different runs of the GA optimiser. S4 Figs. 1 to 4 illustrates the model predictions for these four runs. As it can be seen the predictions and their general behaviours are similar then the model and fitting regime are robust from multiple GA runs’ point of view. Additionally, to make a quantitative comparison between the four fittings, final MSE values—as defined in supplementary material (S1)—for S4 Figs 1a to 4a approximately became, respectively, 0.104, 0.105, 0.103 and 0.104 for 500 fitting points.

**S4 Table 1**: Parameters for the mathematical P2X model with respct to different GA optimiser runs.

| Parameter | Run 1 | Run 2 | Run 3 | Run 4 |
| --- | --- | --- | --- | --- |
| $\boldsymbol{\alpha}_{\boldsymbol{1}\boldsymbol{x}\boldsymbol{7}}$ | 2418.502 | 2013.856 | 3002.345 | 2998.519 |
| $\boldsymbol{\alpha}_{\boldsymbol{2}\boldsymbol{x}\boldsymbol{7}}$ | 1483.681 | 1764.069 | 1243.537 | 1246.121 |
| $\boldsymbol{\alpha}_{\boldsymbol{3}\boldsymbol{hx}\boldsymbol{7}}$ | 0.03030 | 0.03544 | 0.0248 | 0.0248 |
| $\boldsymbol{\beta}_{\boldsymbol{1}\boldsymbol{hx}\boldsymbol{7}}$ | 2.182 | 10.574 | 3.805 | 3.683 |
| $\boldsymbol{\beta}_{\boldsymbol{2}\boldsymbol{hx}\boldsymbol{7}}$ | 2.612 | 2.613 | 2.614 | 2.616 |
| $\boldsymbol{\beta}_{\boldsymbol{3}\boldsymbol{hx}\boldsymbol{7}}$ | 0.0125 | 0.01206 | 0.0126 | 0.0126 |
| $\boldsymbol{\beta}_{\boldsymbol{4}\boldsymbol{hx}\boldsymbol{7}}$ | 997.0402 | 1004.905 | 1006.398 | 779.098 |

**(b)Q )**

**(a)Q )**

| 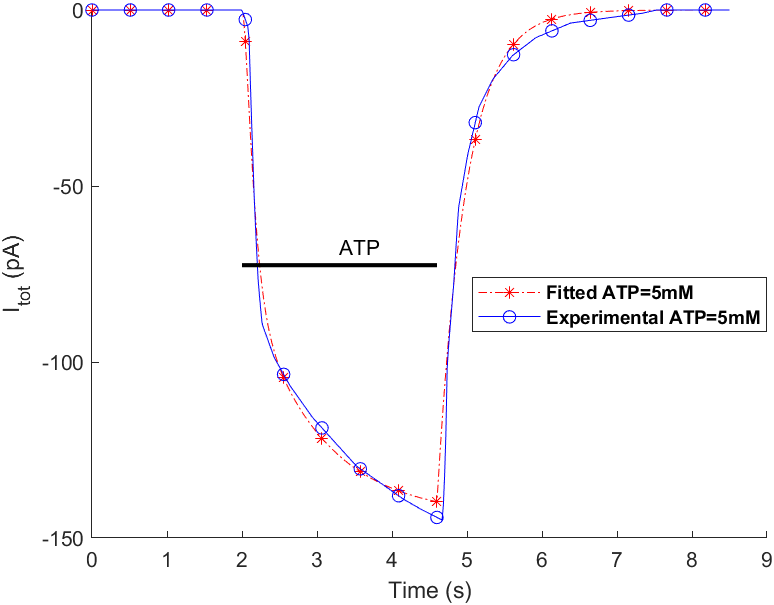 | 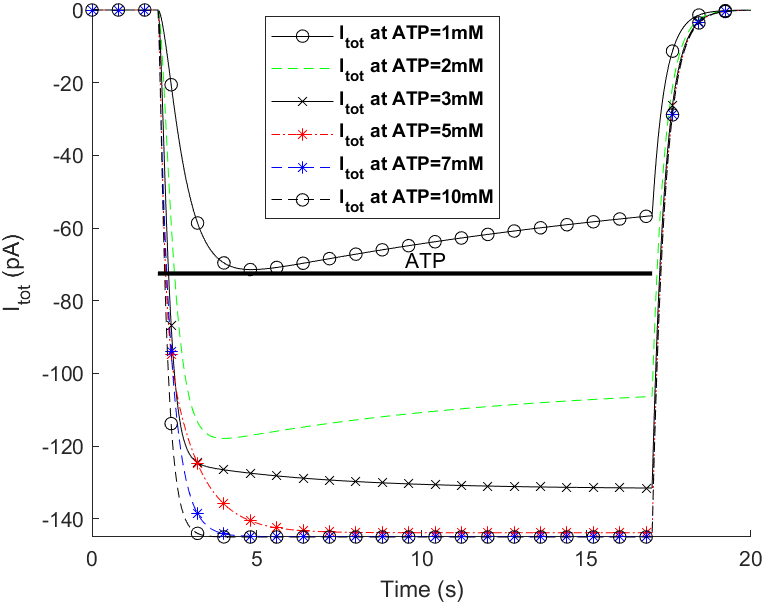 |
| --- | --- |

**S4 Fig 1**: Running the model fitting for the first time: (a) Fitting of the P2X model, and (b) model predictions.

| 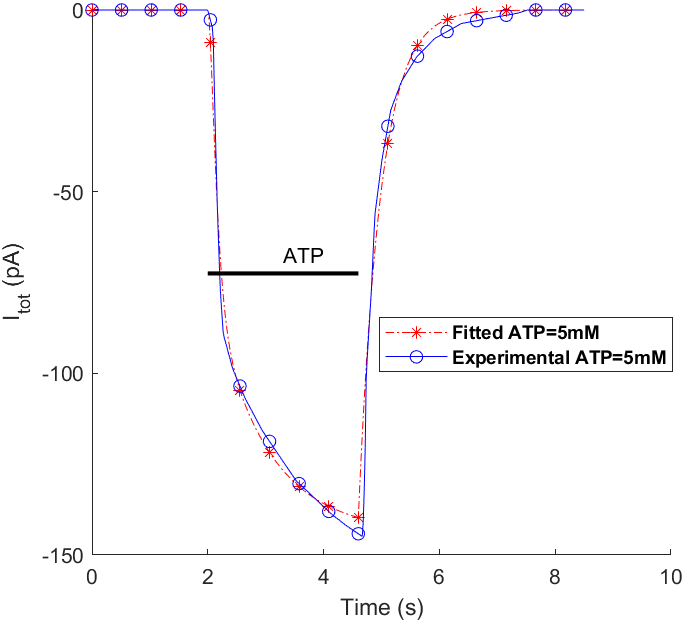 | 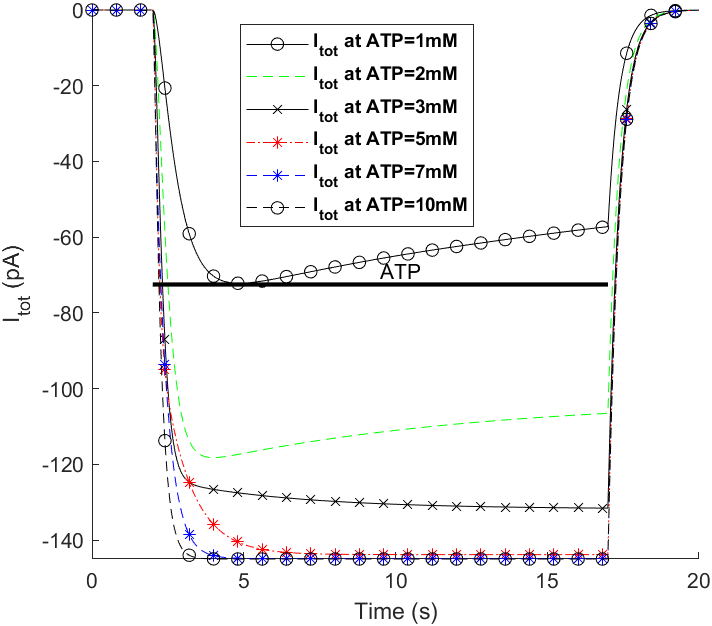  **(b)Q )** |
| --- | --- |

**S4 Fig 2**: Running the model fitting for the second time: (a) Fitting of the P2X model, and (b) model predictions.

**(a) )**

| 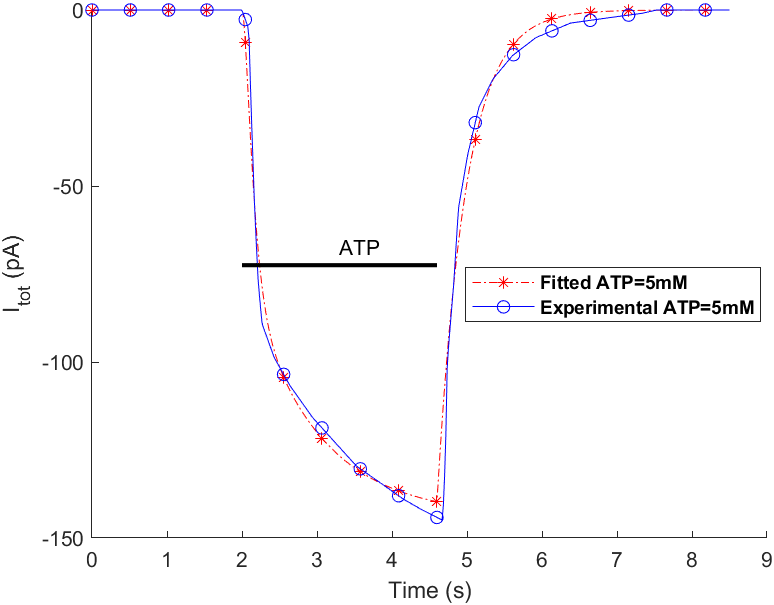  **(a) )** | 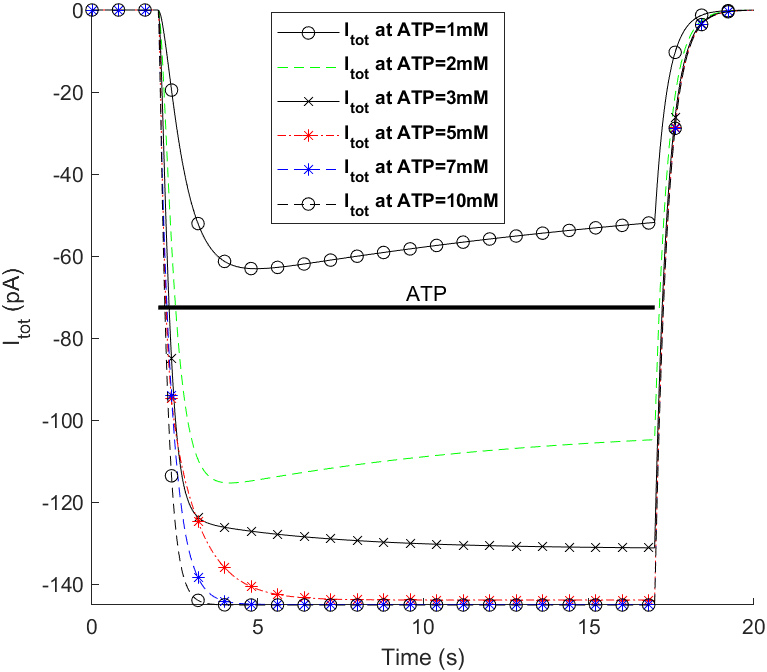  **(b)Q )** |
| --- | --- |

**S4 Fig 3**: Running the model fitting for the third time: (a) Fitting of the P2X model, and (b) model predictions.

| 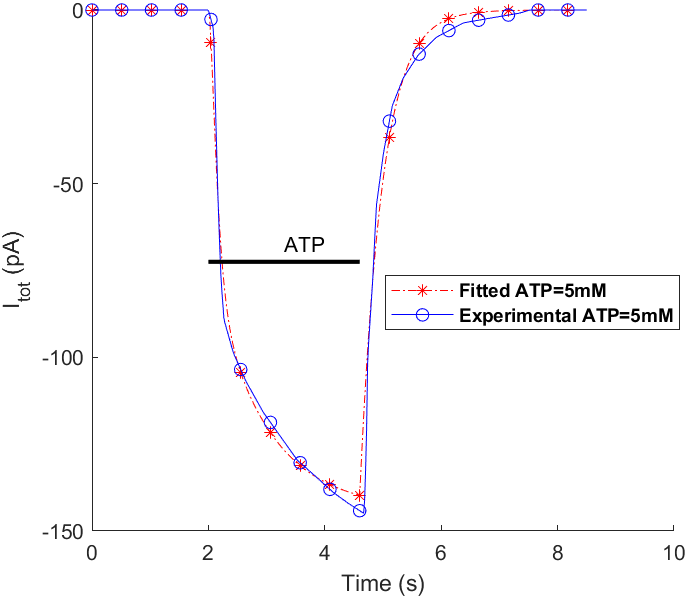  **(a) )** | 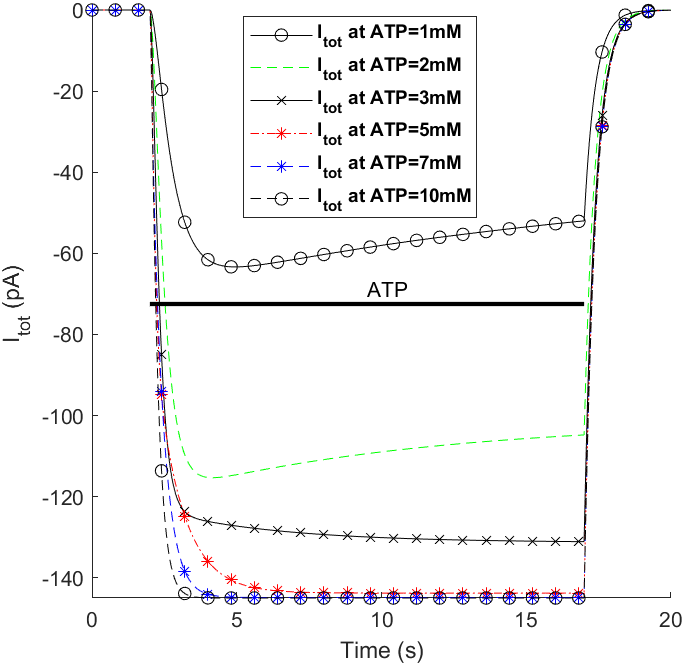  **(b)Q )** |
| --- | --- |

**S4 Fig 4**: Running the model fitting for the fourth time: (a) Fitting of the P2X model, and (b) model predictions.
