# Supplementary material for: Mathematical modelling of human P2X-mediated plasma membrane electrophysiology and calcium dynamics in microglia
Source: PLoS Comput Biol. 2021 Nov 1;17(11):e1009520. doi: 10.1371/journal.pcbi.1009520 (PMC8584768; doi:10.1371/journal.pcbi.1009520)
Supplement: S5 Text — It discusses the necessity of model elements in a quantitative manner. (DOCX) [file pcbi.1009520.s005.docx]

**S5 Text. Contribution of Model Components and Parameters to Represent P2XR Biological Data**

Here, we consider the influence of a number of model components and parameters on fitting and predictions and show that they are necessary to provide a good fit in a try to capture the underlying biological processes. These insights make it clear that the elements of the P2X model in its current form are required for unravelling Ca^2+^ dynamics in human microglia.

**Firstly**, as discussed in the main paper, human P2X_4_R data shows that there are three different phases in the whole-cell patch-clamp current configuration. Let’s assume that $\varphi(A)=A$ is chosen instead of an exponential rate function for P2X_4_R model. S5 Fig 1 illustrates the fitting of the model. As seen, the model response is biphasic and misses capturing the third phase (i.e. deactivation). When $\varphi(A)=e^{\zeta_{1}A}$ is used, the model is able to capture the third phase mainly due to $\varphi(A)$ becoming one when agonist is removed (namely, the path S → O does not disppaer during deactivation phase and allows the model to make use of its corresponding link). This exponential function (which depends on ATP amplitude) is also effective to capture the overall properties of observed current at the high level of ATP in human monocyte-derived macrophages[1]. Because the the model has exponential dependnecy on agonist, all model parameters are optimised for two and three phases of the experimental data simultaneously. This is one of the main reasons that allows the model to make use of fewer state variables in contrast to the existing P2X models in the literature as given in the main paper.

| 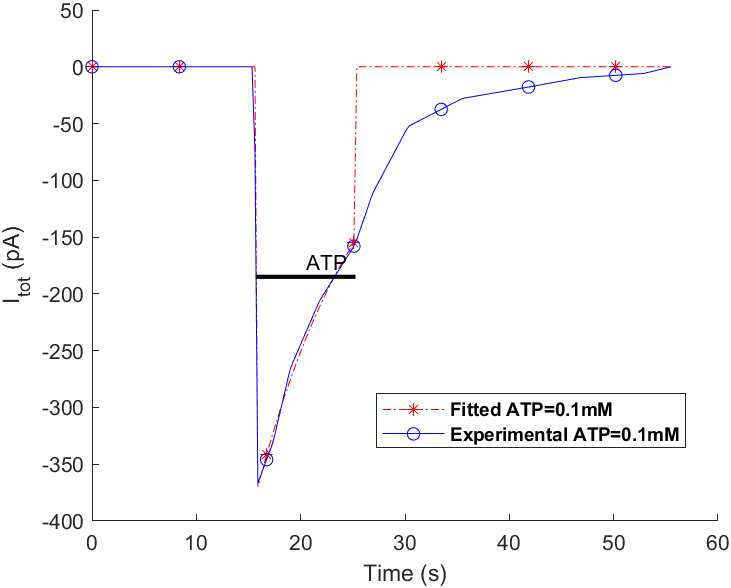 | 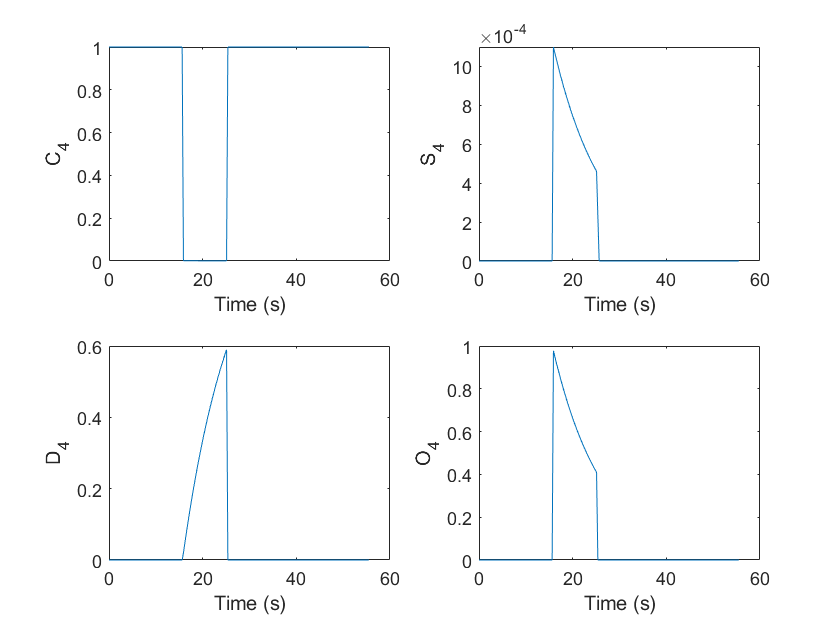 |
| --- | --- |

**S5 Fig 1**: Fitting of the P2X_4_R model using a linear rate as function of agonist for $\varphi(A)=A$.

**Secondly**, as dicsussed in the main paper, the kinetic rates of the $S\rightleftarrows D$ transition is chosen as an exponential form in order to control/create biphasic/triphasic behaviours in the model outputs. As a test, let’s remove the transition from S to D by setting $\beta_{34}=0$ in the P2X_4_R model. S5 Fig 2 shows that the model fitting is not good and is also unable to capture the triphasic shape of the experimental data.


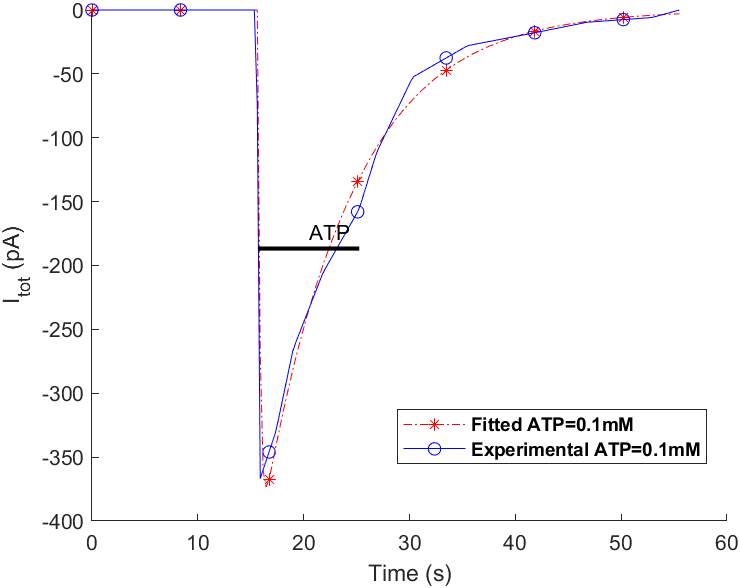


**S5 Fig 2**: (a) Fitting of the hP2X_4_R model when $\beta_{34}=0$.

**Thirdly**, choosing linear backward rates where there is no exponential dependency on agonist gives rise to a very bad fit that is certainly useless. S5 Fig 3 shows that such a choice makes the model fail to capture the experimental data correctly.


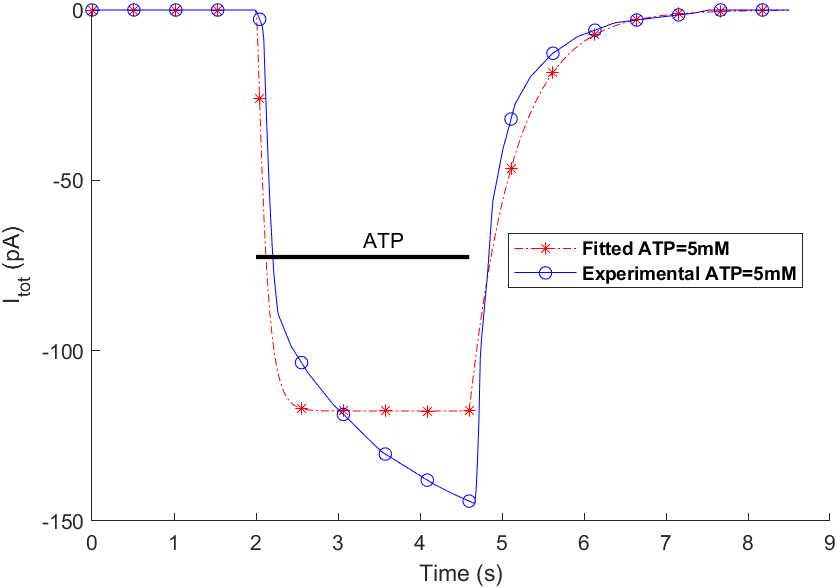


**S5 Fig 3**: Fitting of the P2X_7_R model when backward rates of S → C and O → S are constant, namely, $\beta_{14}$ and $\beta_{24}$.

**Fourthly**, we allowed the optimiser to find the value of $\zeta_{1}$. The fitting quality is shown in S5 Fig 4 which is not satisfactory. Hence, $\zeta_{1}$ was estimated manually by setting different values for $\zeta_{1}$ and letting the optimiser to find other parameters.

**
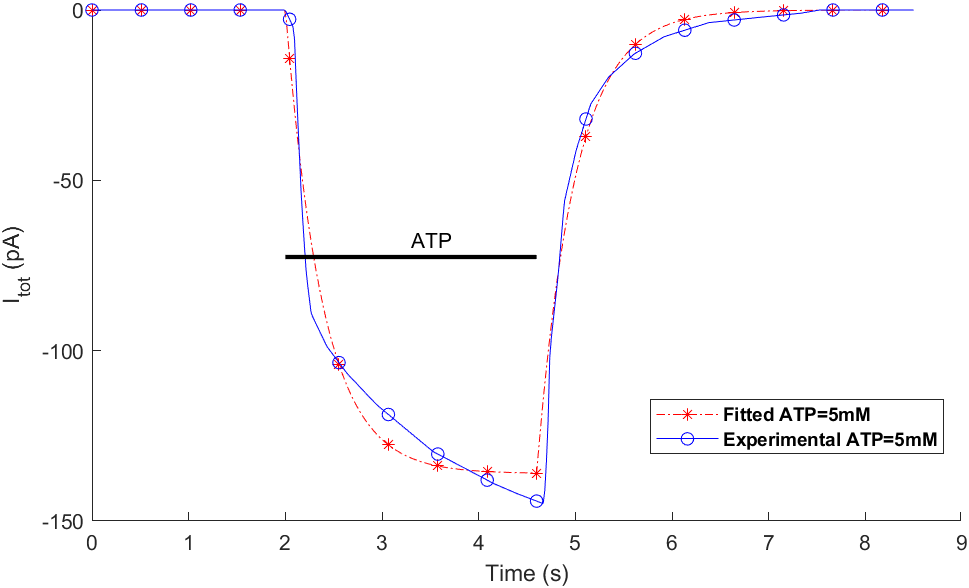
**

**S5 Fig 4**: Fitting of the P2X_7_R model when the optimser is allowed to find the value of $\zeta_{1}$.

**Fifthly**, as explained in the main article, the exponential rate function of $e^{{-\zeta}_{2}O}$ in D → C transition is functionally used for guaranteeing the state D returns to its resting state. This function is realised because the value of the exponential rate term becomes one when O state goes to zero. To graphically show this fact, we removed the link between D to C in the P2X_7_R model. S5 Fig 5 shows the transient response and the model states after fitting. It is obvious that D state cannot be depleted and C state also does not go to 1 whereas S and O states stabilise finally. So, D → C transition cannot be removed from the model. It is worth noting that much more states that give rise to a complicated model is needed to deal with such an issue if one wants not to use the exponential form of kinetic rates proposed by our P2X model.

| 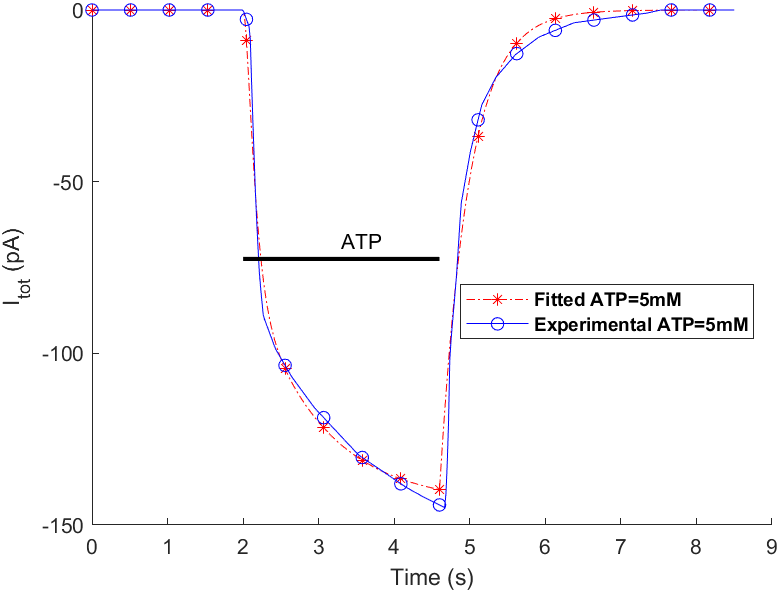 | 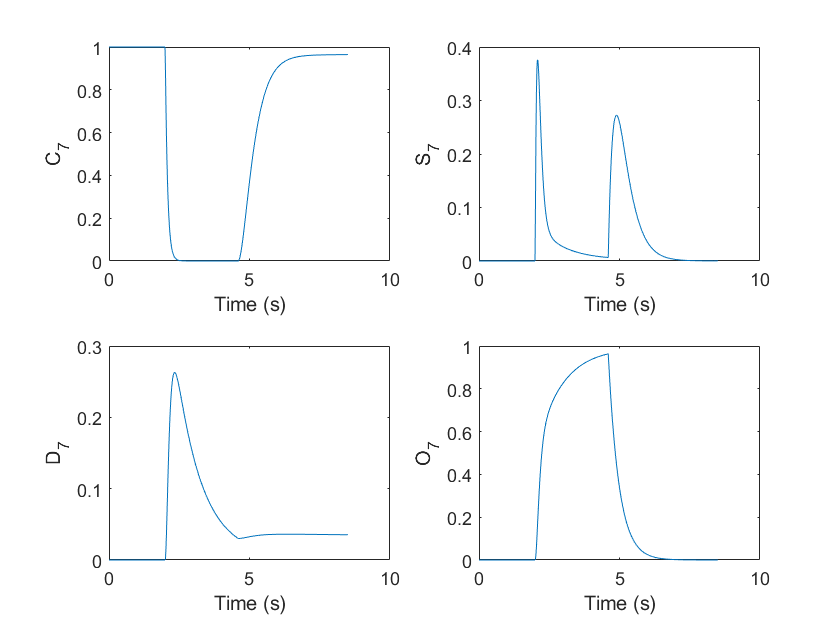 |
| --- | --- |

**S5 Fig 5**: Transient response (left panel) and state space (right panel) of the fitted total current upon ATP treatment via activation hP2X_7_ receptor in where D state is eliminated from the model.

**Sixthly,** as ponited out in the main paper, the $S\rightleftarrows D$ transition and its exponential rate functions are an integral part of the model to support different patterns of biphasic/triphasic gating properties of P2XRs. We removed the D state from P2X_7_R model to see how it affects the fitting. S5 Fig 6 shows that the model failed to capture the activation phase closely providing that D state is eliminated from the model. Initial guess of large or small values of rates for this transition for both P2X models was chosen such that the GA optimiser can succeed in good fits. Furthermore, S5 Fig 7 illustrates that both fitting and predictions are monophaisc for hP2X_4_ receptor when D state is removed from its corresponding model. Also, in both cases, the C state does not return to its resting state after the removal of ATP (not shown).

**
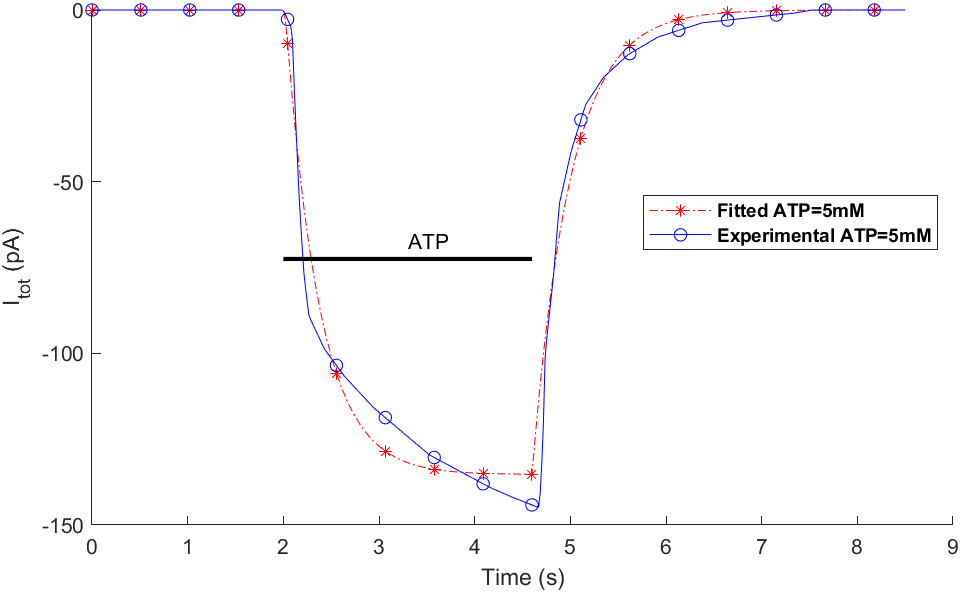
**

**S5 Fig 6**: Transient response of the fitted total current upon ATP treatment via activation hP2X_7_ receptor in where D state is eliminated from the model.

| 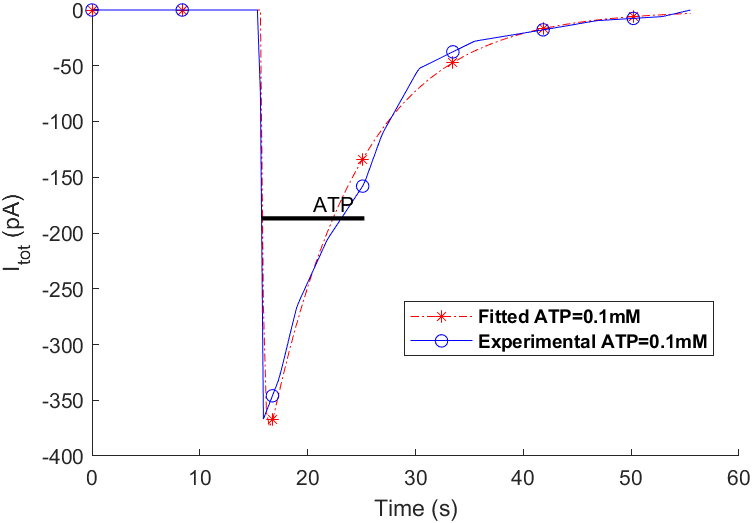  **(a)** | 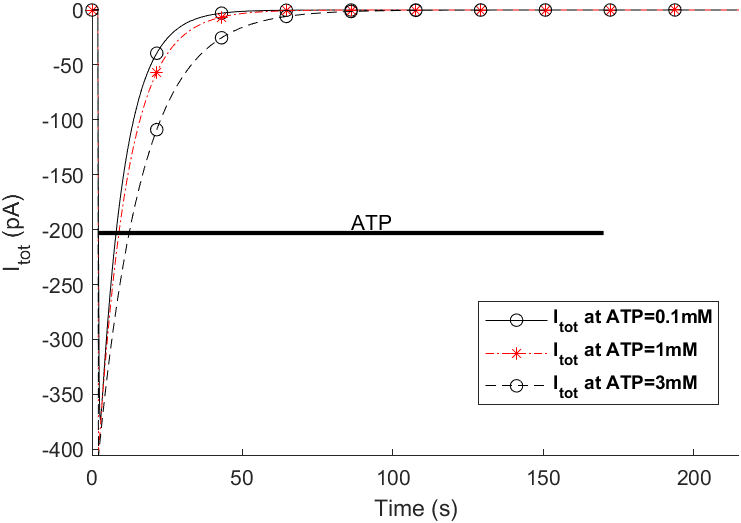  **(b)** |
| --- | --- |

**S5 Fig 7**: Transient responses of the fitted total current (a) and predictions (b) upon ATP treatment via activation hP2X_4_ receptor in where D state is eliminated from the model.

**Seventhly,** as dicussed in the the main text, a careful treatment was made to estimate $\zeta$ parameters in the model. Mainly, we used a trial-and-error strategy to manualy find optimal values for $\zeta$ parameters and to let the optimiser find all remaining parameter values. To show this quantitatively, we ran the optimiser with, for example, setting$\zeta_{1}=5\times{10}^{3}$. As illustrated in S5 Fig 8, fitting is by no means satisfactory by choosing this specific value for $\zeta_{1}$.

**
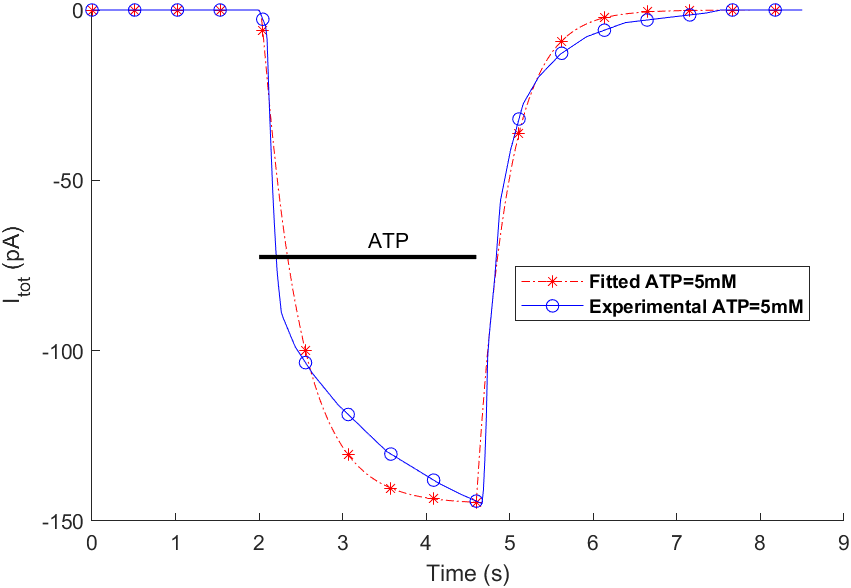
**

**S5 Fig 8**: Transient response of the fitted total current upon ATP treatment via activation hP2X_7_ receptor by manually choosing$\zeta_{1}=5\times{10}^{3}$.

**Finally,** we conclude that the model is less complex than the existing P2X models in terms of the number of state variables and model parameters. It is minimal with respect to the number of model parameters, because the model cannot be reduced more since its structure is meant to capture drastically different dynamics as a function of agonist, although it may have been made simpler for a single agonist level. As it was dicussed, there is an exponential relationship between kinetic rates and agonist that results in good fits. We believe that similar to the HH formalism for action potentials [2] there should be an elegant exponential relationship between kinetic rates and different levels of ATP. Therefore, this model can be extended when new experimental data becomes available (where the exact form of the rate functions or exact values of$\zeta$ parameters in our model will be determined). It was also shown that every element in the structure of the proposed model (including model parameters and exponential model components) is intended to cope with a specific gating property of P2XRs.

**Supplementary References**

1.Vargas-Martínez EM, Gómez-Coronado KS, Espinosa-Luna R, Valdez-Morales EE, Barrios-García T, Barajas-Espinosa A, et al. Functional expression of P2X1, P2X4 and P2X7 purinergic receptors in human monocyte-derived macrophages. Eur J Pharmacol. 2020;888:173460.

2.Hodgkin AL, Huxley AF. A quantitative description of membrane current and its application to conduction and excitation in nerve. J Physiol. 1952;117(4):500-44.
