# Supplementary material for: Mathematical modelling of human P2X-mediated plasma membrane electrophysiology and calcium dynamics in microglia
Source: PLoS Comput Biol. 2021 Nov 1;17(11):e1009520. doi: 10.1371/journal.pcbi.1009520 (PMC8584768; doi:10.1371/journal.pcbi.1009520)
Supplement: S6 Text — It provides a better illustration of multiple sensitives given in Fig 15. (DOCX) [file pcbi.1009520.s006.docx]

**S6 Text. Separate Graphs for Local Sensitivity Analysis of the Model**

Because there are 18 relative sensitivities in Fig 15, they are shown again separately in S6 Fig 1 to S6 Fig 9 for a better illustration. Note that corresponding parameters for P2X_7_R and P2X_4_R models are organised in a single figure. For example, sensitivities due to conductances come together in S6 Fig 2.


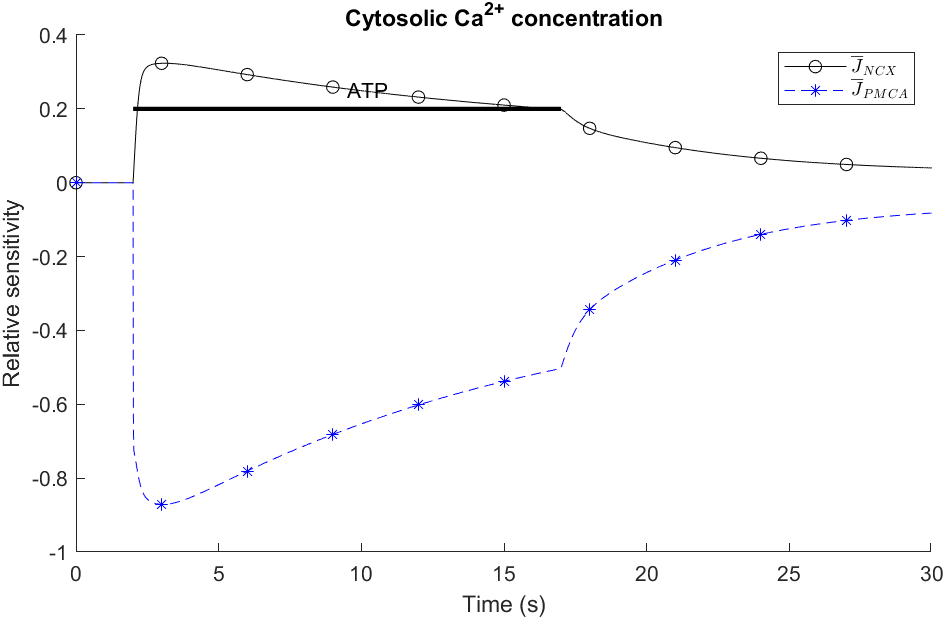


**S6 Figure 1**: Relative sensitivities for $\bar{J}_{PMCA}$ and $\bar{J}_{NCX}$.


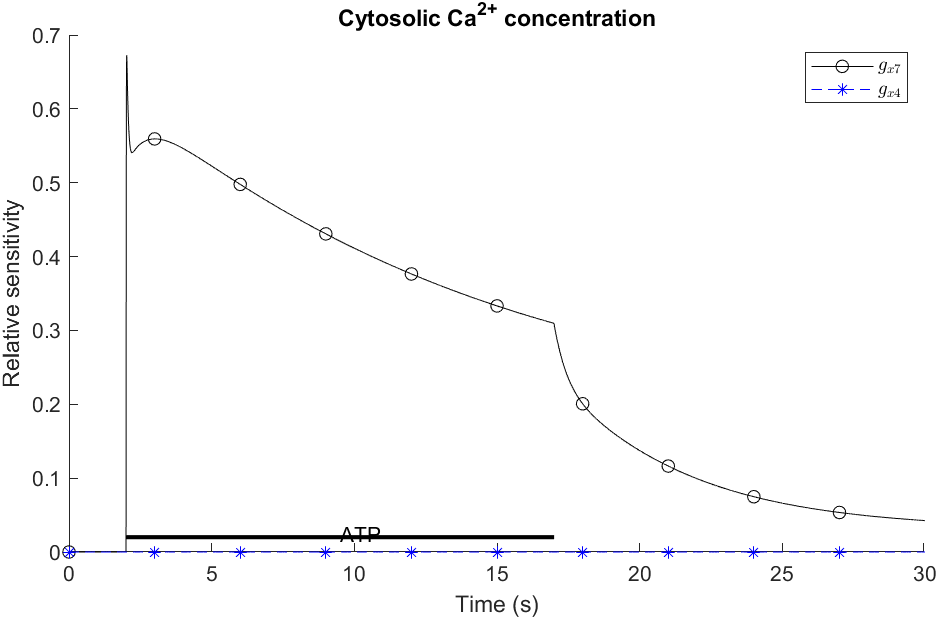


**S6 Figure 2**: Relative sensitivities for $g_{x7}$ and $g_{x4}$.


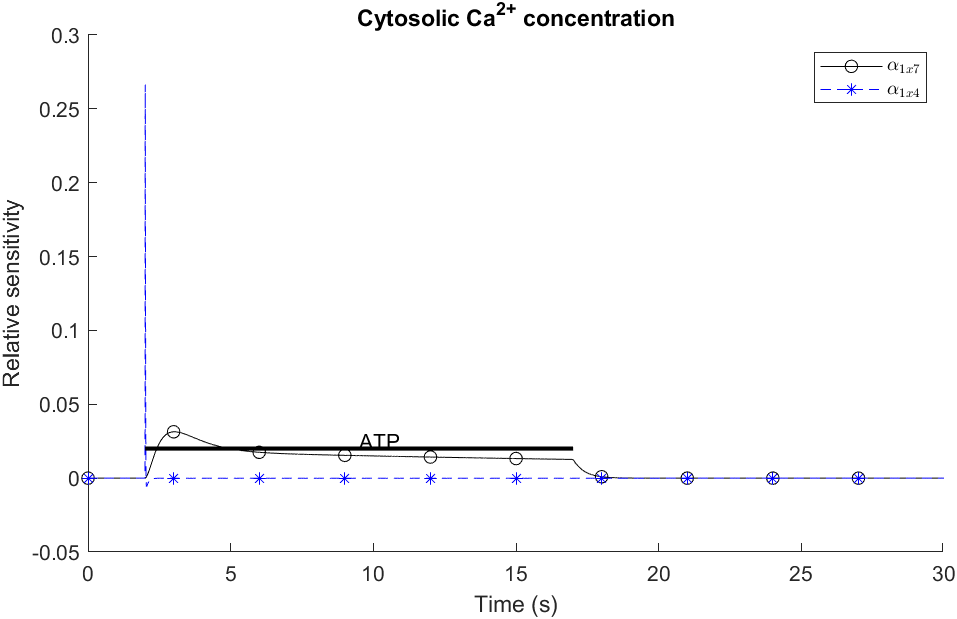


**S6 Figure 3**: Relative sensitivities for $\alpha_{1x7}$ and $\alpha_{1x4}$.


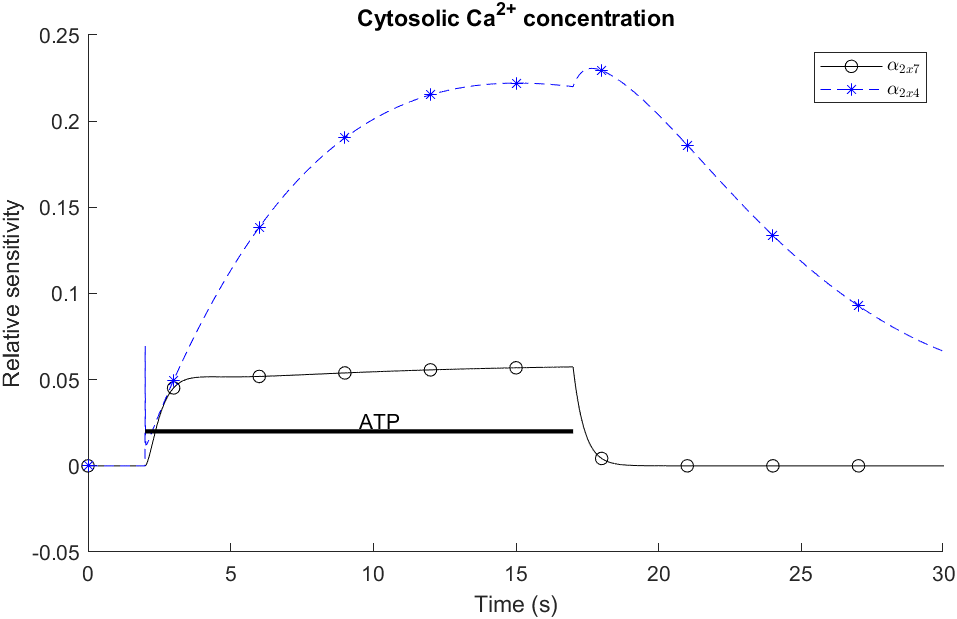


**S6 Figure 4**: Relative sensitivities for $\alpha_{2x7}$ and $\alpha_{2x4}$.


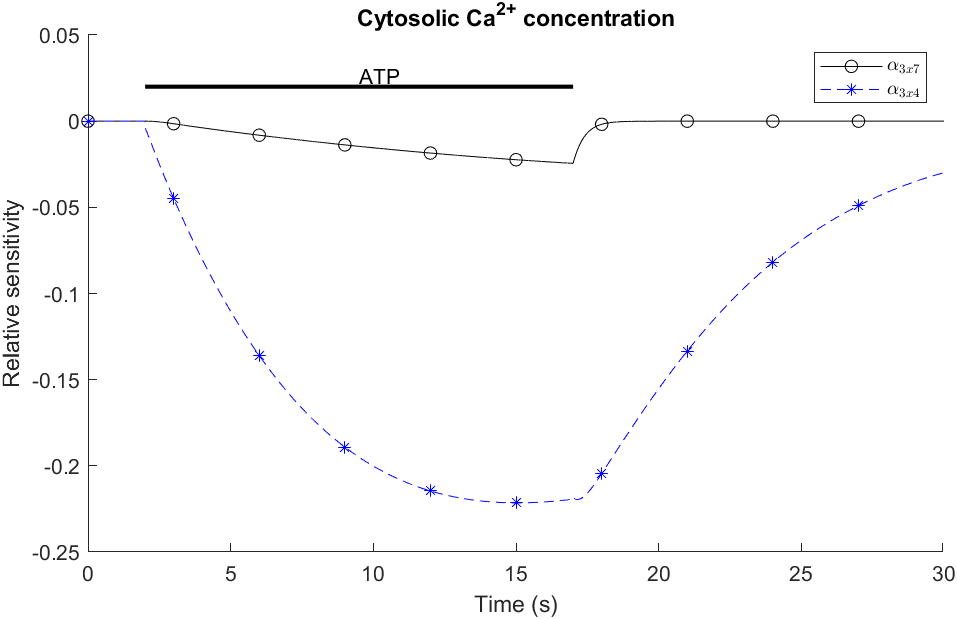


**S6 Figure 5**: Relative sensitivities for $\alpha_{3x7}$ and $\alpha_{3x4}$.


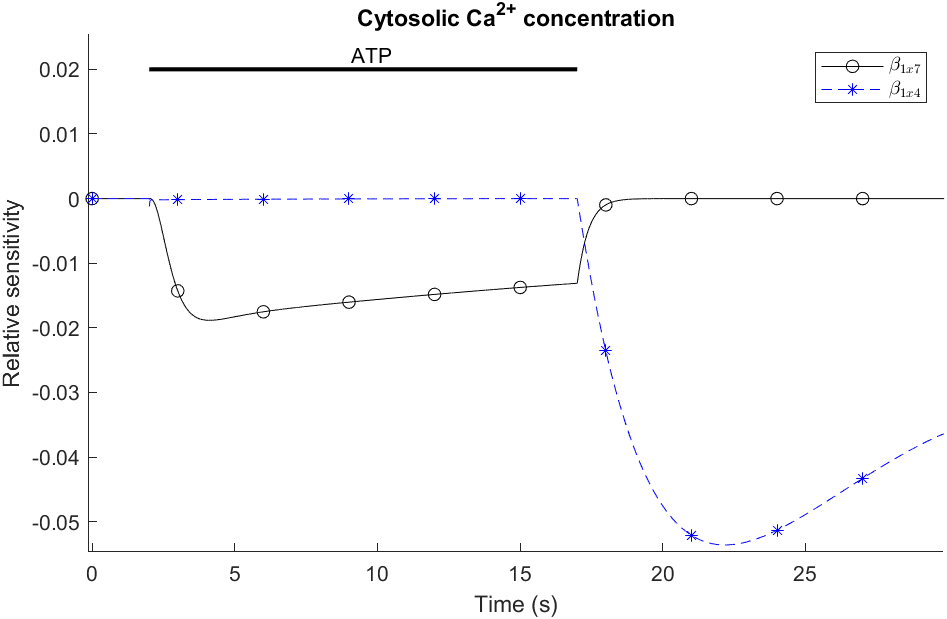


**S6 Figure 6**: Relative sensitivities for $\beta_{1x7}$ and $\beta_{1x4}$.


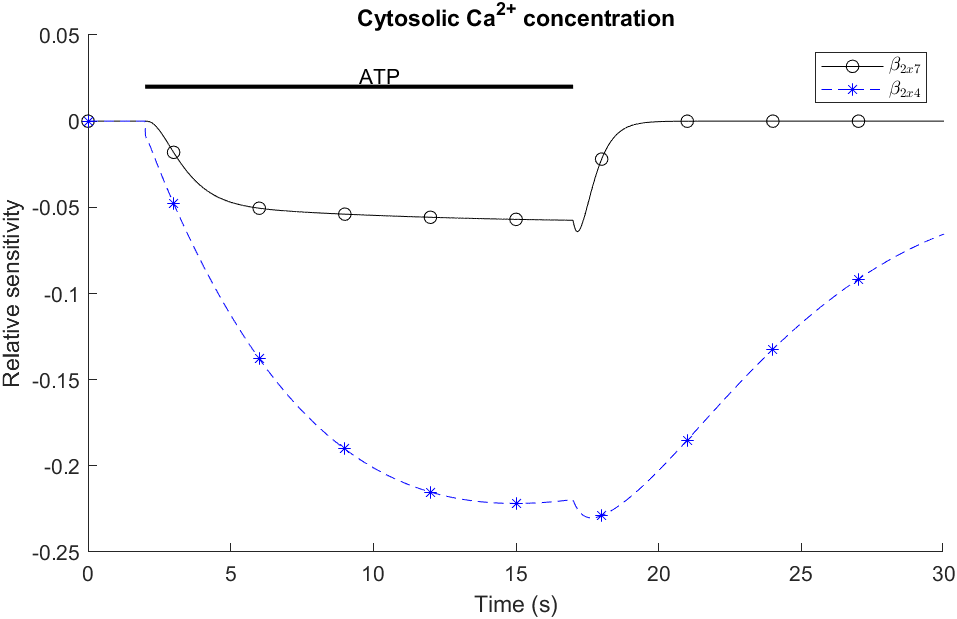


**S6 Figure 7**: Relative sensitivities for $\beta_{2x7}$ and $\beta_{2x4}$.


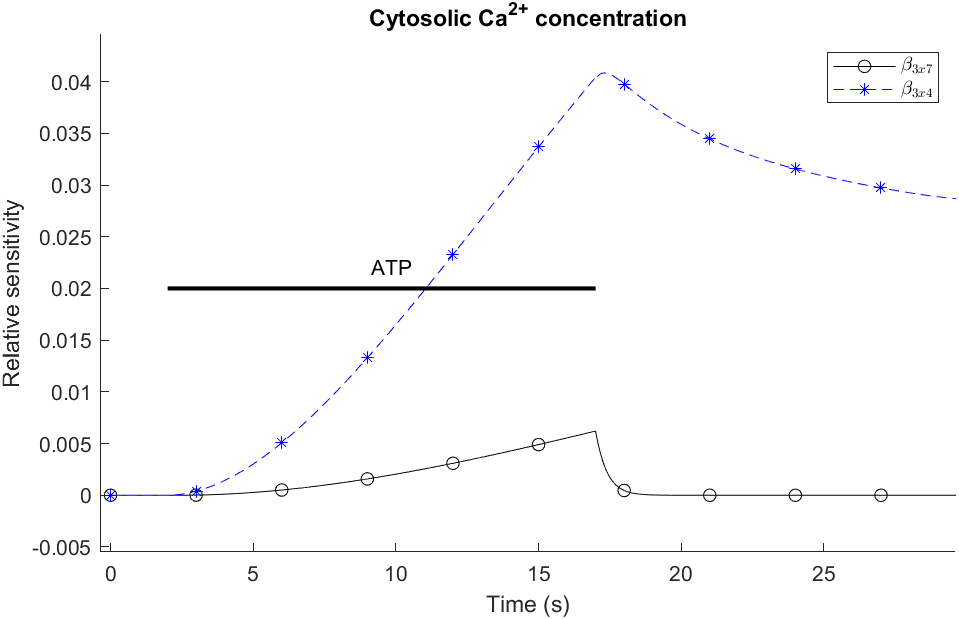


**S6 Figure 8**: Relative sensitivities for $\beta_{3x7}$ and $\beta_{3x4}$.


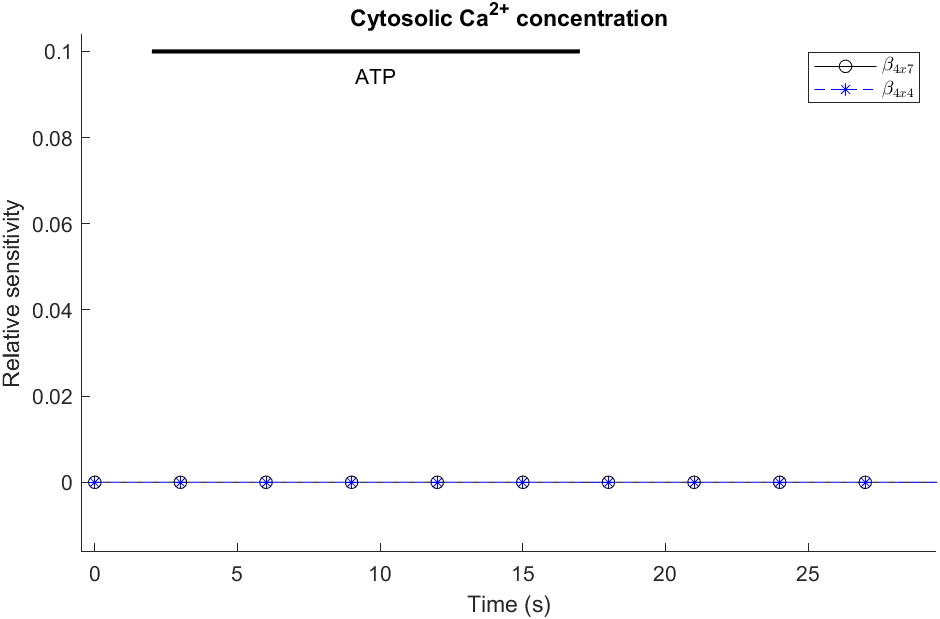


**S6 Figure 9**: Relative sensitivities for $\beta_{4x7}$ and $\beta_{4x4}$.
